# Resonant Inelastic X‑ray Scattering: How Well Does LR-TDDFT Perform?

**DOI:** 10.1021/acs.jpca.5c04528

**Published:** 2025-09-17

**Authors:** Erik Vitols, Vinícius Vaz da Cruz, Thomas Fransson, Iulia Emilia Brumboiu

**Affiliations:** † Faculty of Physics, Astronomy and Informatics, 414324 Nicolaus Copernicus University in Toruń, 87-100 Toruń, Poland; ‡ Division of Theoretical Chemistry and Biology, KTH Royal Institute of Technology, 100 44 Stockholm, Sweden; § Helmholtz-Zentrum Berlin für Materialien und Energie, Institute for Methods and Instrumentation for Synchrotron Radiation Research, Albert-Einstein-Strasse 15, D-12489 Berlin, Germany; ∥ Independent Researcher, Stockholm 111 64, Sweden

## Abstract

Resonant inelastic X-ray scattering (RIXS) is one of the most information-rich spectroscopic techniques, uniquely capable of probing excited states through their dependence on momentum, energy, and polarization. However, the inherent difficulty in interpreting RIXS signals underlines the critical need for accurate and efficient spectral calculations to facilitate their understanding. While hierarchical wave function-based methods such as the algebraic diagrammatic construction (ADC) scheme for the polarization propagator have shown promising results in the calculation of RIXS spectra, they remain computationally demanding. To enable fast and efficient RIXS calculations, we evaluate the performance of linear-response time-dependent density functional theory (LR-TDDFT). Two LR-TDDFT approaches are investigated: the restricted-subspace approximation, which uses a subset of occupied and virtual orbitals to compute the valence-excited and selected core-excited states at the same time, and the two-shot LR-TDDFT approach, where the core- and valence-excited states are calculated independently. We benchmark a range of functionals—including global hybrid, range-separated, and tailored range-separated variants—on a set of small molecules using ADC as the reference. We find that all range-separated hybrids and most global hybrid functionals exhibit good agreement with the reference across all metrics. Furthermore, we demonstrate the applicability of LR-TDDFT by computing the RIXS spectrum of C_60_.

## Graphical abstract

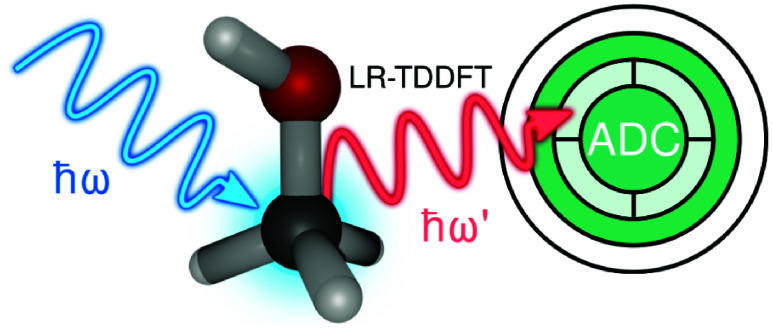

## Introduction

Resonant inelastic X-ray scattering^1,2^ (RIXS) spectroscopy is considered one of the most sophisticated and information-rich spectroscopic probes of the properties of matter. This status derives from several unique properties: (1) transferring the site and chemical sensitivity of core-level spectroscopy to the manifold of valence excitations;^3,4^ (2) enabling the investigation of vibrational excitations and the mapping of ground-state potential energy surfaces^5–7^ with sensitivity to hydrogen bonding;^8–10^ (3) probing collective excitations, e.g., phonon, magnon, and plasmon dispersions in solids;^11–14^ and (4) exhibiting quantum interference phenomena.^15–17^

RIXS is a coherent two-photon process; in a simplified picture, an incident X-ray photon excites the system into a transient core-excited state, which subsequently decays onto a manifold of valence-excited states, emitting a secondary photon—or, in the case of nonradiative scattering, an Auger electron. Core–core RIXS, where the final-state manifold consists of lower energy core-excited states,^2,3,11^ can also be considered,^18^ but the focus here is on valence-excited final states. RIXS can further be divided into direct and indirect scattering.^1,19^ In direct RIXS, the core-excited electron remains in the valence-excited orbital, while another electron annihilates the core-hole (CH). In indirect RIXS, a core electron is excited into a high-lying orbital, and the resulting CH induces nonadiabatic valence excitations—known as shake-ups^2^—as the electrons dynamically screen the CH potential,^19^ before the originally excited electron recombines with the CH. Direct RIXS, synonymous with resonant X-ray emission (RXES), is the dominant channel when dipole allowed—as is the case for the K edge of light elements^2^—and will be the focus of this study. RIXS, as a second-order process,^2^ is not subject to the same selection rules that govern one-photon spectroscopies. In solution, RIXS can therefore resolve solute states otherwise obscured by solute–solvent interactions.^20^

Large efforts have been devoted to push the experimental limits of the technique both in terms of spectral resolution and to extend its applicability to molecular problems in complex chemical environments. Examples of radiation facilities which have dedicated RIXS beamlines are the Veritas beamline at the MAX IV laboratory (Sweden),^21^ the Heisenberg RIXS instrument at the European X-ray Free Electron Laser (XFEL) (Germany),^22^ or the qRIXS and chemRIXS endstations of the Linear Coherent Light Source (LCLS, United States). The interpretation of RIXS signals, however, is far from trivial and relies heavily on quantum chemical calculations. Historically, RIXS has often been modeled via a decoupled two-step procedure, where the absorption and emission are treated independently. An example is the work by Fouda et al., who determined RIXS spectra of gas-phase water using two separate calculations of X-ray absorption spectroscopy (XAS), respectively, XES transition energies and transition dipole moments.^23^ In response methods, such as algebraic diagrammatic construction (ADC), linear response (LR) or equation-of-motion (EOM) coupled-cluster (CC), or linear-response time-dependent density functional theory (LR-TDDFT), XAS is typically calculated by making use of the core valence separation (CVS) approximation.^24^ XES, instead, can be modeled starting from a core-ionized reference where the relevant valence-to-core transitions appear with negative energies.^25–29^ Other approaches proposed for their very low computational cost involve using transition energies and associated transition moments obtained directly from the independent-particle Kohn–Sham DFT orbitals.^30,31^

Treating the scattering as a one-step process instead leads to the Kramers–Heisenberg–Dirac (KHD) formalism of RIXS, which includes channel interference and polarization anisotropy. A nonexhaustive list of approaches within this formalism include the Green’s function based ADC for the polarization propagator in the intermediate state-representation (ISR) using the complex polarization propagator (CPP) method, by Rehn et al.^32^ Similarly, RIXS scattering amplitudes have been implemented at the damped linear response coupled-cluster (CC) theory level,^33,34^ as well as by using the CVS approximation.^35^ Multireference methods have also proven successful—for example, restricted active space self-consistent field^36,37^ (RASSCF) and the density matrix renormalization group^38^ (DMRG), the latter applied to solids, restricted to systems with quasi-one-dimensional character. LR-TDDFT-based approaches include the restricted-subspace approximation (RSA),^39^ where only selected core, occupied, and virtual orbitals are used to solve a response equation with reduced size which returns the core-excited and valence-excited states of interest. Additionally, LR-TDDFT has been used in a two-shot (2S) setup, where the core-excited and valence-excited states are determined separately.^40,41^ We emphasize that the restricted subspace approximation defined above is not the same as the core–valence separation approximation. In the context of RIXS, RSA involves freezing a set of occupied (donor) and unoccupied (acceptor) orbitals to be able to obtain both the core-excited and valence-excited states in one single LR-TDDFT calculation. On the other hand, the CVS approximation consists of neglecting the coupling between core- and valence-excited states and diagonalizing a much smaller response matrix involving only excitations from core orbitals. The CVS approximation therefore allows to obtain the core-excited states alone. The terminology in the literature can be somewhat confusing, so we would like to stress this distinction between RSA and CVS.

RIXS involves both core-excited and valence-excited states, so benchmarks that evaluate the performance of various methods for core-excitations and valence-excitations may be useful, but not necessarily a priori applicable also to RIXS. Presumably, both types of states should be described at a similar accuracy level, so independent benchmarks can still provide a preliminary idea of how different methods are expected to perform. In this respect, the current “gold standards” for XAS and core-excited states of medium-sized organic molecules are considered to be the extended second order CVS-ADC(2)-x and EOM-CCSD^42^ methods. For XES, ADC(2)-x was also found to yield the most accurate results for relative features within the ADC hierarchy.^26,27^ Similarly for valence-excited states, the ADC and CC methods stand as the gold standard for medium-sized organic molecules. ADC(3) and its ADC­(3/2) variant were found to perform the best in the ADC hierarchy for excitation energies and excited state properties,^43,44^ where the ADC(2)-x variant was found to perform worse than ADC(2). On the other hand, CC3 performed best in the CC hierarchy for excitation energies with very small mean absolute errors.^45,46^ Compared to CC, ADC has the advantage of being Hermitian, simplifying the derivation and implementation of transition density matrices and excited-state observables.^47^

In spite of the proliferation of theoretical methods to simulate RIXS, systematic theoretical benchmarks of the most commonly used techniques are still lacking. Recent efforts addressed this issue for X-ray absorption spectroscopy,^42^ however extending this effort to RIXS remains a significant challenge. The purpose of this paper is to address this deficiency by investigating, evaluating and thus benchmarking the performance of adiabatic linear-response TDDFT-based approaches, using various exchange-correlation (xc) functionals. Since direct comparisons of calculated data to experimentally measured RIXS spectra pose numerous challenges (see Results and discussion), the reference is here chosen to be ADC. For this purpose, we implemented the 2S approach at the ADC level, i.e. obtaining the core-excited states from a CVS-ADC(2)-x calculation and the valence-excited states from ADC(3), where the expressions for the state-to-state transition density matrices at these two theory levels are formally equivalent. Finally, earlier studies^25,39,41,48,49^ of RIXS using LR-TDDFT have all employed the Tamm–Dancoff approximation (TDA), while here we implement instead the transition density matrices for full LR-TDDFT. In the present work we restrict ourselves to vertical (fixed-nuclei) electronic excitations.

## Theory

### Resonant Inelastic X-ray Scattering

As a second-order process, RIXS is described by second-order time-dependent perturbation theory,^50^ which in the resonant regime yields the double differential scattering cross-section in terms of the Kramers–Heisenberg–Dirac amplitudes:^1,2,50–54^

$$\frac{d^{2}\sigma \left(\omega ,\omega^{\prime }\right)}{d\Omega_{k\prime }d\omega^{\prime }}{|}_{\mathrm{res}}={\left(\frac{r_{e}}{m_{e}}\right)}^{2}\frac{\omega^{\prime }}{\omega }\underset{f}{\sum }|\underset{n}{\sum }\frac{\left\langle f\right|\varepsilon^{\prime }\cdot \hat{p}\left|n\right\rangle \left\langle n\right|\varepsilon \cdot \hat{p}\left|g\right\rangle }{\hbar \omega -\left(E_{n}-E_{g}\right)+i\Gamma_{n}/2}{|}^{2}\delta \left(\omega^{\prime }+\omega_{fg}-\omega \right)$$ 1

with the system (molecule) initially in the electronic ground state |*g*⟩. Here, *m*_e_ denotes the electron mass and *r*_e_ the classical electron radius. The expression describes the scattering of an incident photon with energy *ℏ*ω, wave vector ***k***, and polarization vector **ε** into a solid angle element dΩ_***k*** ′_, within the energy window *ℏ* [ω′, ω′ + dω′], where the scattered photon is characterized by (***k*** ′, **ε**′). An idealized narrow-band incident photon beam is assumed, with spectral function δ­(ω).^55,56^ We omit the Thomson scattering term, as well as the off-resonant contribution arising from the alternate time ordering—where emission precedes absorption.^1,50^ The electric dipole approximation *e* ^–*i*(***k***–***k*** ′)· ***r̂***_*i*_^ ≃ 1 has been assumed, valid for core-level spectroscopy of light elements.^24,57^ *E*_*n*_ is the eigenvalue of eigenstate |*n*⟩ of the molecular Hamiltonian.^58^ Γ_*n*_ denotes the full-width-at-half-maximum (fwhm) lifetime broadening of the intermediate state |*n*⟩, which is taken to be element-dependent but constant across all intermediate states *n*.^59,60^ Final-state broadening is neglected (Γ_*f*_ ≪ Γ_*n*_), leading to the appearance of the delta function δ­(ω′ + ω_*fg*_ – ω), obtained in the narrow-Lorentzian limit, which ensures energy conservation of the overall process.^15^ When the energy separation between the relevant intermediate states is large compared to Γ_*n*_, the off-diagonal terms in eq 1—which encode the interference—can be discarded.^1,15,61^ Furthermore, since Γ_*n*_ is only present in the intermediate states this suggests that the core-excited state broadening is *not* a limit for the experimental resolution, allowing for subnatural line-width resolution.^1,15,19^ Inclusion of nuclear degrees of freedom—omitted in the present work—can be essential for achieving agreement with experiment, particularly for molecules with dissociative core-excited states, such as the extensively studied water-,^6,8,36,62^ and methanol^63^ molecules. Nonetheless, the aim of this study is to provide a benchmark for vertical RIXS, which often yields a qualitatively correct spectral description compared to experiment.^32,39,40^

In contrast to adsorbed molecules with well-defined orientations, molecules in the liquid- or gas-phase are randomly oriented and so the cross-section must be averaged over all molecular orientations. Additionally, experiments rarely measure the polarization of the scattered photon; typically, only the outgoing wave vector ***k*** ′ is recorded. Hence, for a specific final state *f*, we average over all molecular orientations, as well as over all emission polarization vectors **ε**′, of eq 1, yielding^1,15^

$$\sigma_{f\leftarrow g}\left(\omega ,\theta \right)={\left(\frac{r_{e}\hbar^{2}}{m_{e}}\right)}^{2}\frac{\omega^{\prime }}{\omega }\frac{1}{15}\underset{\xi ,\xi^{\prime }}{\sum }\left[\left(2-\frac{1}{2}\sin^{2}\left(\theta \right)\right)F_{fg}^{\xi \xi^{\prime }}{\left(F_{fg}^{\xi \xi^{\prime }}\right)}^{*}+\left(\frac{3}{4}\sin^{2}\left(\theta \right)-\frac{1}{2}\right)\left(F_{fg}^{\xi \xi^{\prime }}{\left(F_{fg}^{\xi^{\prime }\xi }\right)}^{*}+F_{fg}^{\xi \xi }{\left(F_{fg}^{\xi^{\prime }\xi^{\prime }}\right)}^{*}\right)\right]$$ 2

where ξ, ξ′∈{*x*, *y*, *z*} and θ is the angle between the incident photon polarization vector and the scattered photon propagation direction, **ε** · ***k*** ′/ ∥ ***k*** ′∥ = cos­(θ). The Kramers–Heisenberg–Dirac scattering amplitude tensor, $F_{fg}^{\xi \xi^{\prime }}$ , is written^51,52^

$$F_{fg}^{\xi \xi^{\prime }}\left(\omega \right)=\frac{1}{\hbar }\underset{n}{\sum }\omega_{fn}\omega_{ng}\frac{\left\langle f\right|{\mathrm{\mu ̂}}_{\xi }^{†}\left|n\right\rangle \left\langle n\right|{\mathrm{\mu ̂}}_{\xi^{\prime }}\left|g\right\rangle }{\omega -\omega_{ng}+i\Gamma_{n}/\left(2\hbar \right)}$$ 3

The commutation relation [*Ĥ*, ***r̂***] = −*i* ***p̂*** has been used to write the electric dipole operator in the length gauge, **μ̂** = −**r̂**. We have introduced the resonance frequency *ℏ*ω_*ng*_ = *E*_*n*_ – *E*_*g*_. eqs 2 and 3 are our final working equations for the spectral calculations. The sum over states *n* formally spans *all* intermediate states |*n*⟩, including the continuum—indicative of the difficulty, if not impossibility, of evaluating such an amplitude.^2^ While truncation is unavoidable in practice, the dominance of the resonance ensures that reducing the full manifold of intermediate states {|*n*⟩} to a subset close to the resonance introduces only a negligible error.^64^

### Linear-Response TDDFT

Within ground-state linear response theory, the excitation energies ω_*ng*_ appear as the poles of the frequency-dependent response function, and the corresponding residues yield the transition moments. This prescription, starting from a DFT ground state, leads to the linear-response TDDFT equations,^65–67^ analogous to those of the random phase approximation^68^ (RPA),

$$\left(\begin{array}{ll} A & B\\ B^{*} & A^{*} \end{array}\right)\left(\begin{array}{l} X^{n}\\ Y^{n} \end{array}\right)=\omega_{ng}\left(\begin{array}{ll} 1 & 0\\ 0 & -1 \end{array}\right)\left(\begin{array}{l} X^{n}\\ Y^{n} \end{array}\right)$$ 4

The matrix elements and further details are given elsewhere.^65–67,69^ The excitation- and de-excitation vectors, {|**X** ^*n*^, **Y** ^*n*^⟩} satisfy biorthonormality as induced by the metric in eq 4.^68^ The Tamm-Dancoff approximation (TDA) to eq 4 is obtained by letting **B** →**0**, whereby the eigenvalue problem reduces to the Hermitian form^66,68,70^ **AX** ^*n*^ = ω_*ng*_ **X** ^*n*^.

eq 3 shows that transition dipole moments between excited states are needed. Linear response theory describes the *ground-state* density response to a perturbation; thus to describe transitions between excited states, we formally require quadratic response^71^ (QR). However, in addition to the greatly increased computational cost of QR-TDDFT, it has been shown that, due to the adiabatic approximation which neglects the frequency dependence of the exchange and correlation kernel, one may obtain spurious transitions when the transition energy between two excited states matches that of any one excited state.^72–74^ For illustrative examples of how this issue can cause divergences, e.g., in the derivative coupling vectors, we direct the reader to refs 75 and 76 . See, for example, Figure 4 in ref 75 or Figures 5 and 6 in ref 76. Instead, we will approximate the second-order matrix elements at first order, which has proven successful in the case of excited-state absorption^77^ (ESA), nonadiabatic couplings,^73,78,79^ and previous applications of LR-TDDFT to RIXS.^25,39,40^ The concept of obtaining state-to-state transition dipole moments within a linear-response TDDFT framework is often known as the pseudowave function approximation (PWA),^40,78^ where excited-state (pseudo-) wave functions are determined in formalisms which do not have a static wave function ansatz.^80–82^ Through second quantization, the transition dipole moments can be expressed in terms of the one-particle transition density matrix (1TDM).

### Reduced Transition Density Matrix

The transition dipole moments in eq 3 are written

$$\left\langle f\right|{\mathrm{\mu ̂}}_{\xi }^{†}\left|n\right\rangle =\underset{p,q}{\sum }\left\langle \phi_{p}\right|{\mathrm{\mu ̂}}_{\xi }\left|\phi_{q}\rangle f|{â}_{p}^{†}{â}_{q}\right|n\rangle =\underset{p,q}{\sum }\mu_{pq}^{\xi }\gamma_{pq}^{fn}$$ 5

with ξ ∈{*x*, *y*, *z*}, and *â*_*p*_^†^(*â*_*p*_) denoting the creation (annihilation) operator—creating (annihilating) a particle in orbital *p*. The quantity γ_*pq*_^*fn*^ is identified as the one-particle transition density matrix (1TDM) between the excited states *f* and *n*. Since we will be dealing with purely electric dipole perturbations, in the length gauge, and real-valued wave functions, we can assume the excitation vectors to be real,^65^ (**X** ^*n*^)^†^ = (**X** ^*n*^)^T^, ∀*n*. The 1TDM between excited states is, in TDA, given by

$$\gamma_{pq,\mathrm{TDA}}^{fn}=\{\begin{array}{l} \gamma_{ij}^{fn}=-\underset{a}{\sum }X_{ia}^{f}X_{ja}^{n},\\ \gamma_{ab}^{fn}=\underset{i}{\sum }X_{ia}^{f}X_{ib}^{n},\\ \gamma_{ai}^{fn}=\gamma_{ia}^{fn}=0, \end{array}$$ 6

while the 1TDM from the ground state is simply the corresponding excitation vector

$$\gamma_{pq,\mathrm{TDA}}^{ng}=\{\begin{array}{l} \gamma_{ai}^{ng}=X_{ia}^{n},\\ \gamma_{ab}^{ng}=\gamma_{ij}^{ng}=\gamma_{ia}^{ng}=0. \end{array}$$ 7

Here, *i*, *j*, ··· index occupied molecular orbitals (MOs), and *a*, *b*, ··· index unoccupied MOs.

General operator transition elements between two excited states, or between the ground state and an excited state, can, in the equation-of-motion formalism,^83^ be obtained via commutator algebra, from which the transition density matrices can be identified.^79,84^ For RPA/LR-TDDFT the 1TDM between two excited states *f* and *n* is obtained from^79,84^

$$2\left\langle f\right|{â}_{p}^{†}{â}_{q}\left|n\right\rangle =\left\langle g\right|\left[{Ĉ}_{f},\left[{â}_{p}^{†}{â}_{q},{Ĉ}_{n}^{†}\right]\right]-\left[\left[{â}_{p}^{†}{â}_{q},{Ĉ}_{f}\right],{Ĉ}_{n}^{†}\right]\left|g\right\rangle$$ 8

with the excitation operator

$${Ĉ}_{n}^{†}=\underset{ia}{\sum }\left(X_{ia}^{n}{â}_{a}^{†}{â}_{i}-Y_{ia}^{n}{â}_{i}^{†}{â}_{a}\right)$$ 9

This leads to

$$\gamma_{pq,\mathrm{RPA}}^{fn}=\{\begin{array}{l} \gamma_{ij}^{fn}=-\underset{a}{\sum }\left(X_{ia}^{f}X_{ja}^{n}+Y_{ia}^{f}Y_{ja}^{n}\right),\\ \gamma_{ab}^{fn}=\underset{i}{\sum }(X_{ia}^{f}X_{ib}^{n}+Y_{ia}^{f}Y_{ib}^{n}),\\ \gamma_{ai}^{fn}=\gamma_{ia}^{fn}=0. \end{array}$$ 10

The ground state–excited state 1TDM is determined as

$$\left\langle g\right|\left[{Ĉ}_{n},{â}_{p}^{†}{â}_{q}\right]\left|g\right\rangle =\gamma_{pq,\mathrm{RPA}}^{ng}=\{\begin{array}{l} \gamma_{ai}^{ng}=X_{ia}^{n},\\ \gamma_{ia}^{ng}=Y_{ia}^{n},\\ \gamma_{ij}^{ng}=\gamma_{ab}^{ng}=0. \end{array}$$ 11

### LR-TDDFT Approaches to RIXS

Accessing both valence- and core-excited states within a single LR-TDDFT calculation essentially requires a full diagonalization of the underlying response matrix. However, an efficient computation of RIXS amplitudes requires avoiding such full-spectrum treatments. To this end, we investigate two general LR-TDDFT approaches: the restricted-subspace approximation (RSA) by Vaz da Cruz et al.,^39^ and the method of Nascimento et al.,^41^ here referred to as the two-shot (2S) approach.

The 2S approach constructs the RIXS amplitudes using two independent LR-TDDFT calculations. One calculation is performed within the CVS approximation,^24^ in which the coupling between core-excitations and valence-excitations is neglected, allowing the diagonalization of a smaller response equation involving only single-excitations from the core-orbitals of interest. The effect of the CVS approximation on XAS has been investigated elsewhere^85,86^ and the errors introduced are generally small. The number of excited states included determines the number of intermediate states for RIXS. Similarly, a standard valence LR-TDDFT calculation is carried out which defines the final-state manifold, {|*f*⟩}. The results of these two calculations are then combined to form the 1TDMs between two excited states, as per eq 10, while the ground-to-core-excited state transition moments are obtained solely from the CVS calculation eq 11. Note that the CVS excitation vectors are padded with zeros in the corresponding valence-occupied blocks to rescale the CVS problem back to the full size of the Hamiltonian. It should be added that the CVS approximation implies that the excitation vectors from the two independent LR-TDDFT calculations are eigenvectors of different Hamiltonians, i.e., they will no longer necessarily be orthogonal, which may introduce small errors.

The restricted-subspace approximation^39^ (RSA), on the other hand, yields all required response properties from a single LR-TDDFT calculation. The approximation includes only restricted occupied and virtual subspaces, retaining the core orbitals of interest, and those valence-occupied and virtual orbitals deemed essential for describing valence-excited states. While this requires only a single calculation, it comes at the expense of a loss in “black-boxness”—in that there is no known a priori principle for how large the subspaces should be. A balance must be struck: they must be large enough to ensure accuracy, yet small enough to avoid an unmanageable number of excited states. RSA includes some valence–core couplings absent in the CVS-based 2S approach. Since our chosen benchmark molecules are generally small due to the high cost of the ADC reference, we include the full matrix diagonalization of the LR-TDDFT response equations as well, representing the RSA limit with untruncated orbital spaces.

### Algebraic Diagrammatic Construction

The algebraic diagrammatic construction (ADC) scheme for the polarization propagator postulates the existence of a “non-diagonal” form of the polarization propagator^47,87–89^

$$\Pi \left(\omega \right)=x^{†}{\left(\omega Î-\Omega \right)}^{-1}x\overset{\mathrm{ADC}}{=}f^{†}{\left(\omega Î-M\right)}^{-1}f$$ 12

Here, the elements of the diagonal matrix Ω_*n*_ = ω_*ng*_ are the exact vertical excitation energies and *x*_*pq*_^*n*^ = ⟨*n*|*â*_*p*_^†^ *â*_*q*_|*g*⟩ are the transition amplitudes in the basis of exact states {|*n*⟩}. The expansion series for ***f*** and **M**, in the fluctuation potential, are compared with the diagrammatic expansion for the propagator **Π**(ω) and determined through an order analysis. The “diagonal” and postulated “non-diagonal” forms are assumed to be related through a unitary transformation **X**—the solution to the Hermitian secular equation^47^ **MX** = **X** **Ω**, subject to **X** ^†^ **X** = 1. ADC to order *n* [ADC­(*n*)] includes the necessary excitation space and corresponding terms for a consistent description of the propagator to order *n* in its perturbative expansion.

The intermediate state representation^89^ (ISR) of the ADC matrix **M** yields explicit wave functions in the basis of intermediate states {|*J̃*⟩}

$$\left|n\right\rangle =\underset{J}{\sum }X_{J}^{n}\left|\mathrm{J̃}\right\rangle$$ 13

where **X** ^*n*^ is the eigenvector of state *n*. The sum goes over all excitation classes, {*J*}, considered, i.e., singles, doubles, etc., depending on the ADC approximation order. The intermediate states, |*J̃*⟩, are constructed by acting with a set of physical excitation operators, {*Ĉ*_*J*_} = {*â*_*a*_^†^ *â*_*i*_, *â*_*b*_^†^ *â*_*c*_^†^ *â*_*j*_ *â*_*k*_, ···}, on the correlated ground-state wave function, |MP*n*⟩, followed by orthonormalization. |MP*n*⟩ denotes the *n*th-order Mo̷ller-Plesset (MP) ground state, and using it to derive the intermediate states leads to an ADC scheme of the same order—ADC­(*n*). Hence, ADC­(*n*) can be seen as MP*n* for excited states.^47^ Transition moments between two excited states under a one-particle operator *D̂* can then be written^88^

$$D_{fn}=\left\langle f\right|\mathrm{D̂}\left|n\right\rangle =\underset{I,J}{\sum }X_{I}^{f}\left\langle Ĩ\right|\mathrm{D̂}\left|\mathrm{J̃}\right\rangle X_{J}^{n}=X^{f}\mathrm{D̃}X^{n}$$ 14

where *D̃*_*IJ*_ is a matrix element in the basis of intermediate states. In ISR-ADC­(*n*) the one-particle transition density matrix between two excited states (*f*≠*m*) is given by

$$\gamma_{pq}^{fm\left(n\right)}=\underset{I,J}{\sum }{\left(X_{J}^{f}X_{I}^{m}\langle Ĩ|{â}_{p}^{†}{â}_{q}\left|\mathrm{J̃}\right\rangle \right)}^{\left(n\right)}=\underset{I,J}{\sum }{\left(X_{J}^{f}X_{I}^{m}{\overset{\sim }{\gamma }}_{pq}^{JI}\right)}^{\left(n\right)}$$ 15

where the superscript (*n*) indicates the order in perturbation theory. The derivation and resulting expressions are rather lengthy and are therefore omitted here; see the work by Schirmer and Trofimov^90^ for details.

To obtain the RIXS amplitudes, we adopt a two-shot (2S) approach analogous to that used in LR-TDDFT. Core-excited states are computed using CVS-ADC(2)-x, which treats the doubles block to first order. Valence-excited states are described using ADC­(3/2), which combines ADC(3) eigenvectors with ADC(2) one-particle transition density matrix expressions, for ground-to-excited state transitions. The ADC­(3/2) scheme has been shown to offer significantly improved computational efficiency while introducing only a negligible error compared to the fully consistent ADC(3) approach.^89^ We note that the equations for the 1TDM corresponding to transitions between two excited states are equivalent in ADC(2) and ADC­(3/2).

### Core-Hole Delocalization

The treatment of core-excited states as either localized or delocalized is a key issue in simulations of core-level spectra, particularly in systems with symmetrically equivalent atoms or near-degenerate core orbitals, where the resulting vacancy may naturally delocalize.^24,91^ For RIXS, the problem is all the more crucial owing to the core-excited Jahn–Teller effect^92^ which takes place as a rule, not an exception, for molecular systems that are larger than a diatomic and which possess equivalent chemical sites.^93,94^ This effect causes a strong excitation-energy dependence of the RIXS spectrum and is very sensitive to nuclear dynamics and interference.^1,15,95–97^ Notwithstanding, a reasonable approximation in these scenarios is to use a localized core-hole picture, ignoring interference, when comparing to an experimental spectrum excited on top of the X-ray absorption resonance. Conversely, when comparing to a highly detuned spectrum, a delocalized core-hole picture can be used. For the sake of simplicity, we will throughout this study consider a localized core-hole picture, which has been shown to be optimal for excited-state theories of single-reference character, such as LR-TDDFT with an adiabatic kernel.^94^

## Computational Details

Due to the limitation imposed by the reference ADC method, the benchmark set is restricted to small molecules. The primary set was taken from Yuan et al.,^98^ subject to the condition that the sum of all atomic numbers *Z* < 30 with chemical compositions limited to H, C, N, O, and F. This was complemented with selected numbers 1 through 9 from the XABOOM set,^42^ resulting in a total of 20 O 1s, 35 N 1s, and 43 C 1s edges. The molecular structures were optimized using the cc-pVTZ basis set^99^ at the MP2 ^100^ level of theory with the Q-Chem^101^ version 5.1 software. All LR-TDDFT computations were performed in VeloxChem.^102^ The benchmark calculations employed the aug-cc-pVTZ^103^ basis set for all atoms except the hydrogen atoms, where cc-pVDZ^99^ was used instead, in line with the basis set choice from ref. 42]. A range of exchange–correlation functionals were used, namely: the GGA functional PBE,^104,105^ the hybrid functionals: PBE0,^106^ BHandHLYP,^107^ and M06–2X^108^ (which is a hybrid meta-GGA); the range-separated hybrids: CAM-B3LYP,^109^ the modified rCAM-B3LYP,^110^ CAM-QTP01 ^111^ (with a large fraction of exact exchange in the long-range), ωB97X-D^112^ (with empirical dispersion corrections), and LRC-ωPBEh;^113^ as well as the meta-GGA functionals M06-L^114^ and SCAN.^115^ The evaluation of exchange and correlation (xc) functionals and their derivatives is carried out by VeloxChem through the Libxc library.^116^ The parameters of all xc functionals considered are listed in Table S1 of the Supporting Information. Calculations using time-dependent Hartree–Fock (TDHF) were also included. The functionals were selected to (1) span the relevant rungs of Jacob’s ladder; (2) include those commonly used for excited-state calculations—hence the emphasis on hybrids, and range-separated hybrids; and (3) include those that have proven effective in either core-^42^ or valence-excitation regimes.

Two-shot ADC calculations were carried out with adcc,^117^ starting from a reference state from PySCF.^118,119^ The same basis sets were used as in the LR-TDDFT case. The ADC state-to-state transition density matrices were implemented following the approach developed for time-resolved (TR) X-ray absorption spectroscopy by List and co-workers.^120^ The valence-to-core transition density matrices were computed using a stand-alone in-house Python code which can be obtained from github (https://github.com/iubr/rixs-lr-tddft), or zenodo (10.5281/zenodo.16808911), alongside the optimized geometries of the benchmark molecules. The computed benchmark spectra were broadened with a Lorentzian broadening of 0.6 eV full-width-at-half-maximum (fwhm).

To break the symmetry of delocalized core orbitals in symmetric polyatomic molecules (nine of the molecules included in the benchmark set), we opted to distort the molecular geometry, since this was the only option common to both VeloxChem and adcc in their current implementation. As an example, for the ethylene molecule, the C–H bonds of one of the CH_2_ pairs were stretched by 0.05 Å, a value chosen based on the fact that it is large enough to localize the C 1s orbitals, but small enough not to significantly affect the spectra.^91^ We note, however, that localizing core orbitals by breaking the molecular symmetry is not ideal, as care must be taken for each individual molecule to keep the distortion at a minimum, while still localizing the core orbitals. Therefore, other methods are preferable, such as replacing the core electrons of the symmetry-equivalent noncore-excited atoms by effective core potentials (ECPs), or using a localization procedure such as the Boys localization scheme^121^ or the Pipek-Mezey method.^122^ A comparison between the RIXS spectrum obtained for ethylene using the Boys localization procedure and using the molecular distortion described above is shown in the Supporting Information, Figure S1.

## Statistical Metrics

The spectra are analyzed in energy-loss mode, with the elastic line serving as the reference. This means that the energy positions in the spectra are determined entirely by the valence excitation energies. For the benchmark evaluation, we use the normalized, integrated absolute difference (IAD), shown schematically in Figure 1, as well as the total cross-section ratios of the first spectral features as metrics. The normalized IAD is given by

$$\mathrm{IAD}\left(I,I_{\mathrm{ref.}}\right)=\int_{\omega_{-}}^{\omega_{+}}\left|\frac{I_{\mathrm{ref.}}\left(\omega \right)}{A_{\mathrm{ref.}}}-\frac{I\left(\omega \right)}{A}\right|d\omega$$ 16

over a frequency range [ω_–_, ω_+_], with *A* = ∫_ω–_^ω+^ *I*(ω)­dω. This metric is typically used to analyze the difference between experimental features,^123–130^ but was also proposed for comparing calculated spectra.^131,132^

**Figure 1:**
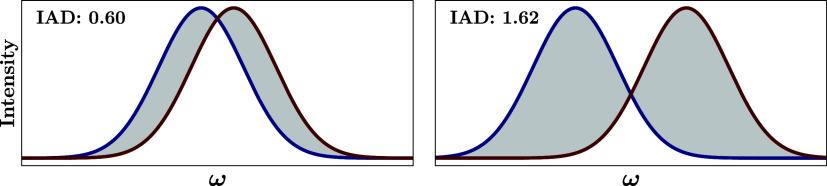
Integrated absolute difference (IAD), given by the gray area, for two equivalent, normalized Gaussians centered at different energies. In the limit of infinite separation (no overlap), the IAD equals 2, as it becomes the sum of the areas of two normalized Gaussians. When the separation goes to zero so does the IAD.

The IAD is agnostic to the origin of the discrepancy, whether it is due to inaccurate spectral features or simply a rigid energy shift—as highlighted in Figure 2. To remedy this loss of information, we introduce the *optimally shifted* IAD, which decouples errors arising from spectral shape and rigid energy shifts. Specifically, we define

$${\mathrm{IAD}}_{\Delta }\left(I,I_{\mathrm{ref.}}\right)=\min_{\Delta }\;\mathrm{IAD}\left(I,I_{\mathrm{ref.}},\Delta \right)=\min_{\Delta }\;\int_{\omega_{-}}^{\omega_{+}}\left|\frac{I_{\mathrm{ref.}}\left(\omega \right)}{A_{\mathrm{ref.}}}-\frac{I\left(\omega +\Delta \right)}{A}\right|d\omega$$ 17

and use the minimizing shift |Δ| alongside IAD_Δ_ as complementary evaluation metrics. Reproducing the correct shape—even with a small rigid shift—is preferable to merely matching the energy range without capturing the spectral features. The optimal shift is defined as the rigid displacement that minimizes the IAD. However, shifting to minimize the IAD without additional constraints can lead to pathological cases, where the shift aligns unrelated features, see Figure 3. In such cases, a smaller shift that does not strictly minimize the IAD may be preferable since the goal is to compare corresponding features. A systematic way of avoiding this issue is to minimize the IAD subject to a penalty term quadratic in Δ, controlled by a parameter λ­[ω^–2^] which sets the strength of the penalty. This would lead to the minimization of a penalized IAD functional of the form:

$${\mathrm{IAD}}_{\Delta ,p}\left(I,I_{\mathrm{ref.}}\right)=\min_{\Delta }\left\{\mathrm{IAD}\left(I,I_{\mathrm{ref.}},\Delta \right)+\lambda \Delta^{2}\right\}$$ 18

In practice, λ can be treated as a tunable parameter, adjusted to balance alignment fidelity with physical plausibility of the applied shift. One can think of $1/\sqrt{\lambda }$ as the energy scale over which shifts are tolerated. We settled for λ = (*E*_–1_^ADC^ – *E*_0_^ADC^)^−1^/4, where *E*_–1_^ADC^ is the energy of the highest-energy peak and *E*_0_^ADC^ the first peak in the spectrum. This value effectively suppresses pathological overshifting while introducing no noticeable artifacts in normal cases. For the few spectra consisting of only one feature, we set λ = 0.

**Figure 2:**
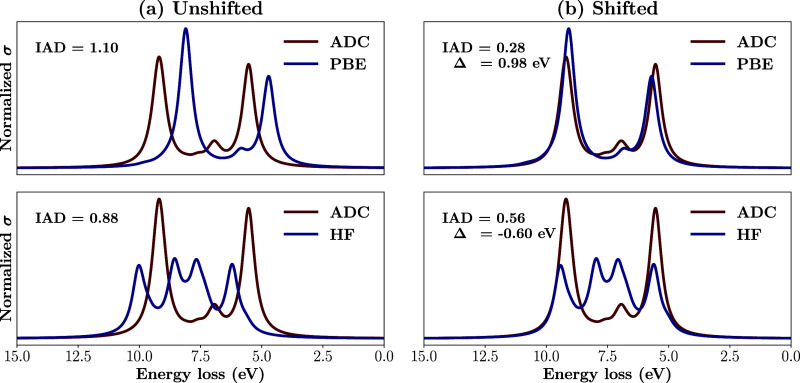
Comparison of unshifted (a) and shifted, by Δ, (b) spectra for PBE and Hartree–Fock (HF). Note that in the unshifted case (a), HF yields a lower IAD than PBE, despite PBE better capturing the spectral features. This becomes evident upon shifting (b), where the IAD for PBE decreases substantially, while the reduction for HF is comparatively smaller.

**Figure 3:**
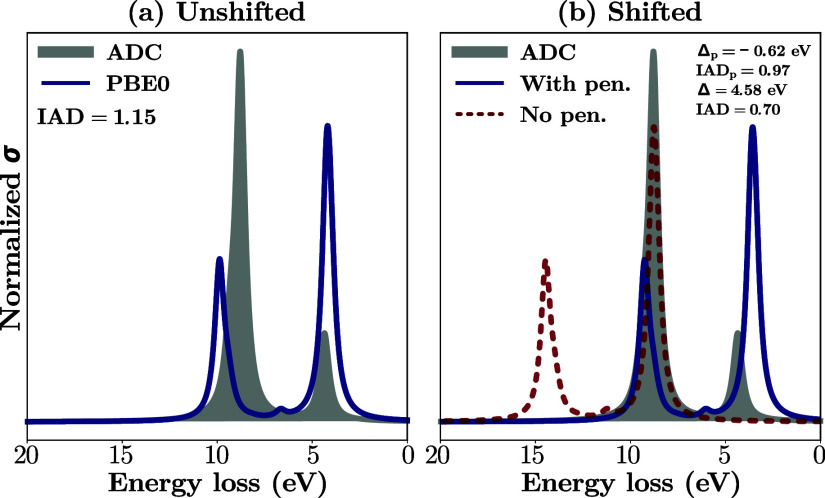
Illustrative example of a pathological shift to minimize the IAD. (a) Unshifted PBE0 spectrum compared to the ADC reference. (b) Shifted PBE0 to minimized the IAD (red dashed line) and shifted PBE0 including a penalty function (blue line). The unrestricted minimization of the IAD incorrectly aligns the first spectral feature of PBE0 with the second feature of ADC. This is remedied by the shift with penalty.

For these metrics, we perform a statistical analysis, reporting the arithmetic mean and corresponding standard error for each edge, method, and functional.

## Results and Discussion

### Validation of ADC as a Reference Method for Electronic RIXS

Here we seek to investigate the performance of LR-TDDFT with different xc functionals in capturing RIXS peak positions and relative intensities over an energy window of several electronvolts (∼10 eV). In this context, benchmarking against experimental RIXS data poses many challenges, even more than for XAS (discussed in detail in ref. 42), XPS,^133^ or XES, since RIXS is much more sensitive to nuclear motion and environment. This means that conformational averaging is typically necessary to capture the experimental features of gas-phase, liquid, and solid state samples. Gas-phase molecules and liquids can exhibit a rich vibrational fine structure which requires wave packet dynamics on the core-excited state potential energy surface (PES) to adequately describe. Apart from these issues, as well as issues related to the experimental setup, calibration, or choice of reference, RIXS measurements can be affected by ultrafast dissociation, e.g., the well-known example of the |1*s* ^–^4*a*_1_⟩ core-excited state of the water molecule.^36^ For these reasons, we opt here for a theoretical reference, where ADC(3) and CVS-ADC(2)-x are, respectively, chosen to describe the valence- and core-excited states involved in the RIXS process.

To validate the performance of 2S ADC(3):CVS-ADC(2)-x, Figure 4 shows measured RIXS spectra of aqueous ammonia,^134^ gas-phase water,^135^ and gas-phase methanol^136^ compared to calculated ADC spectra. The spectra are shown in energy loss, and we note that the calculated data have not been shifted. As a first observation, the positions of the ADC-calculated peaks match quite well the experimental bands. However, some of the relative intensities, especially in the case of water and ammonia, are less well captured. In particular, feature **C** of ammonia and features **B** and **C** in water are greatly overestimated by the calculations. Additionally, features **A** of water and O K-edge methanol present as a double peak in experiment, while only one feature is present in the calculations. Nonetheless, these differences can be explained by several effects that are not considered in our computational model. In the case of water, the splitting of peak **A** into a double peak is explained by ultrafast dissociation, as shown by isotope-dependent measurements,^135^ as well as nuclear wave packet dynamics.^36^ The molecular band is the lower intensity shoulder at lower energy loss, while the sharp peak at higher energy loss (∼ 8 eV) corresponds to the OH^–^ fragment. Furthermore, the RIXS spectrum of the OH^–^ fragment does not display any features in the region of peak B, while contributing in the lower energy loss region of feature C (at ∼ 12 eV).^135^ Another study, using *ab initio* molecular dynamics on the ground and the |1*s* ^–^4*a*_1_⟩ core-excited states of water, found that the position of band **C** is strongly affected by conformational sampling.^23^ Finally, nuclear wave packet dynamics on the |1*s* ^–^4*a*_1_⟩ core-excited state and the |3*a*_1_^–^4*a*_1_⟩ valence-excited state of water, mainly responsible for feature B, showed that the position and intensity of this band is determined by the interplay between ballistic dissociation on the core-excited state PES and a confinement of the wave packet in the Franck–Condon region of the dissociative valence-excited state PES.^137^ These effects explain the lower intensities of features **B** and **C** in the experiment. Similarly, the double peak **A** in the O K-edge RIXS spectrum of methanol is due to ultrafast dissociation on the lowest-energy core-excited state PES, as verified both experimentally^136^ and by nuclear wave packet dynamics.^63^ As in water, the low energy loss shoulder is the molecular band, while the sharp higher energy loss peak corresponds to the CH_3_O^–^ fragment. Finally, the N K-edge RIXS spectrum of ammonia is also affected by dissociation on the lowest core-excited state PES.^134^ The only case unaffected by ultrafast dissociation is the C K-edge RIXS spectrum of methanol where, in fact, the relative intensities of the ADC calculated features match the experiment exceptionally well.

**Figure 4:**
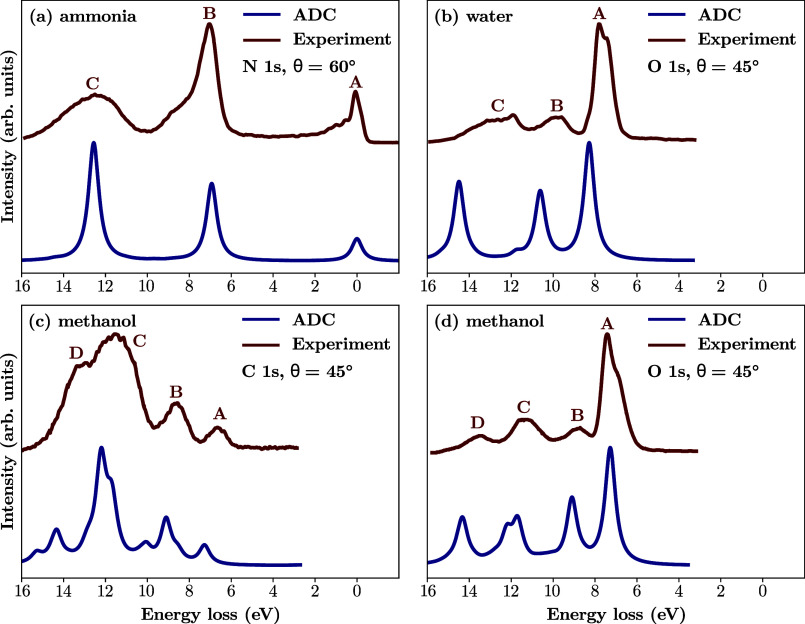
Comparison between RIXS spectra calculated using 2S ADC(3):CVS-ADC(2)-x with the aug-cc-pVTZ basis set and experimental RIXS spectra of (a) aqueous ammonia at the N K-edge,^134^ (b) gas phase water at the O K-edge,^135^ and (c) gas methanol at the C and (d) O K-edges.^136^ The incident photon energy corresponds to the lowest core-excitation and θ denotes the angle between the incident photon polarization and the scattered photon direction. No shift of the calculated data has been performed.

For the benchmark, the 2S ADC(3):CVS-ADC(2)-x approach was used in combination with the aug-cc-pVTZ basis set. This basis set was chosen since it provides a balanced description of both core and valence-excitations, where larger basis sets like aug-cc-pVQZ provided only minimal improvements, but at a much higher computational cost. A comparison of ADC RIXS spectra using different basis sets is included in Figures S2 and S3 of the Supporting Information.

### Benchmark Results

The benchmark results are presented in Figures 5–7; these are the mean shifted integrated absolute difference (IAD), the corresponding mean absolute shift, and the mean intensity ratio of the first feature, with the corresponding standard error. The angle θ between the incoming polarization vector and outgoing photon propagation direction is zero and the incoming photon energy corresponds to the first XAS peak of each molecule. Due to the high computational cost of the ADC reference, the ADC(3) final-state manifold, which is the limiting factor, comprised 20 states for each molecule. The energy loss window used to perform the benchmark was therefore set based on the maximum energy loss value obtained in ADC. The highest energy ADC feature included in the analysis was confirmed by visual inspection of the spectra. To avoid including incomplete RIXS spectral features which would require a higher number of states in the ADC calculations, the highest energy bands in regions with high densities of valence-excited states were discarded. The energy windows obtained this way covered around 10 eV in energy loss for all molecules. The LR-TDDFT valence-excited states were then restricted to cover the same spectral features as in ADC by selecting the last excited state at the same energy as the maximum in the ADC spectrum plus a 0.5 eV padding, to include potential features that lie higher in energy in the LR-TDDFT spectrum.

**Figure 5:**
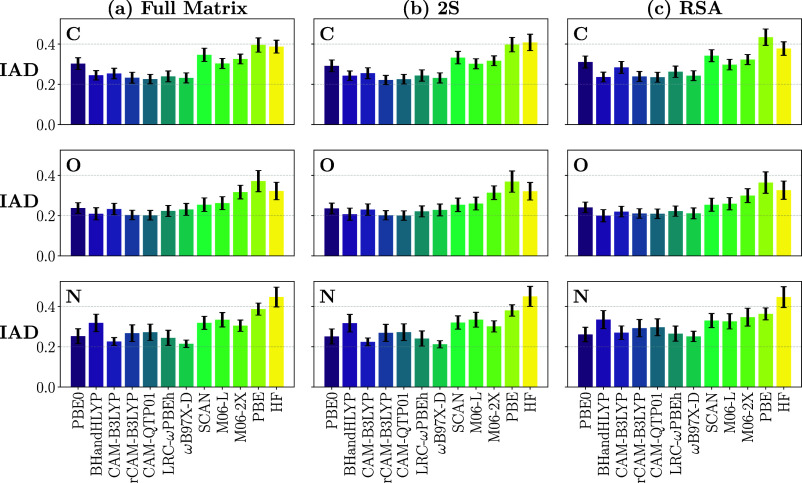
Relative performance of 11 xc functionals and TDHF in predicting RIXS relative peak positions and intensities with respect to ADC for C (top), O (middle), and N (bottom row) K-edges. Relative spectral features are determined in terms of the shifted IAD for (a) full matrix diagonalization, (b) the 2S approach, and (c) RSA.

In Figures 5–7, the three metrics are shown for each approach (full matrix diagonalization, 2S, RSA), for each functional, and for each K-edge studied (oxygen, nitrogen, carbon). To adequately represent RSA in a systematic way, the core orbitals were selected first, based on the desired K-edge. From the remaining occupied orbitals, the 40% lowest energy orbitals were excluded, while the remaining 60% up to the highest occupied molecular orbital (HOMO) were included in the TDDFT response equation, alongside 35% of the virtual orbitals starting with the lowest unoccupied molecular orbital (LUMO). This selection was set based on an investigation of the orbital dependence of RSA, and was deemed striking a reasonable balance between accuracy and efficiency.

In terms of relative features of the RIXS spectra, assessed via the mean shifted IADs in Figure 5, all hybrid functionals considered perform quite well with respect to ADC, where the apparent outliers are, not surprisingly, TDHF and LR-TDDFT using a pure GGA functional (PBE). Slightly worse performances are generally also noted in the case of the M06-L, M06–2X, and SCAN functionals, but not by much. All three edges are reasonably represented, with slightly larger discrepancies in the N K-edge case. Here, we note that some care must be given to using an accurate enough grid for DFT numerical integration, where especially meta-GGA functionals typically require finer grids. Table S1 in the Supporting Information contains the grid details for all the functionals included in this benchmark.

Figure 6 shows the mean absolute energy shifts required to optimally align the LR-TDDFT RIXS spectra to the ADC reference. These shifts are consistently smaller than 0.5 eV for most functionals, except PBE and TDHF, where the shifts are around or above 0.5 eV. Here too, M06-L and SCAN perform slightly worse than the rest. In contrast to the IADs, larger shifts were required for the C and O K-edges, while the N K-edge shifts were similarly low across all hybrids and range-separated hybrids. The consistently small energy shifts across the board may seem surprising considering the large errors in absolute energies, sometimes of the order of 10 eVs, by many xc functionals in the description of core-excited states.^42^ However, we recall that the analysis here is performed in energy loss (*ℏ*ω – *ℏ*ω′), where the error in the ground-state-to-core excitation energy is canceled out by the error in the core-to-valence transition energy. The remaining errors are therefore deemed to be *absolute* errors by the different functionals in capturing valence-excitation energies. In fact, the energy shifts reported here with respect to ADC are quite similar to errors relative to CC2 ^138^ or to accurate experimental values (see ref. 139 and references therein) observed for different DFT functionals in predicting vertical valence-excitation energies.

**Figure 6:**
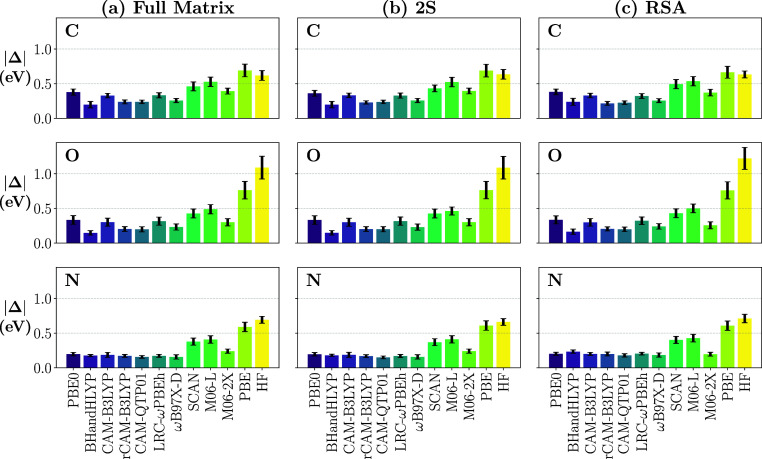
Mean absolute energy shifts in eV required to align RIXS spectra calculated at the LR-TDDFT level with 11 different xc functionals and TDHF to the ADC reference for C (top), O (middle), and N (bottom row) K-edges. LR-TDDFT results obtained with (a) full matrix diagonalization, (b) the 2S approach, and (c) RSA.

Comparing the 2S and RSA approaches to the full matrix diagonalization, their performances in reference to ADC are quite similar, indicating that the errors introduced by these two approximations (with the selected subspace described above in the case of RSA) are small, both in terms of relative spectral features and energies. Comparing the 2S method to RSA, the 2S method appears to perform slightly better but we note that the ADC reference is itself computed within a 2S framework. To further investigate the relative performance of 2S compared to RSA, we repeated the benchmark for all functionals and all metrics, but using the LR-TDDFT full matrix diagonalization as the reference instead of ADC. These results are included in the Supporting Information, Figures S22–S24. The differences between 2S/RSA and full matrix diagonalization are very small, both in terms of relative spectral features, as well as absolute peak positions and intensities. The 2S approach performs better than RSA (with the particular restriction of the orbital spaces made here), where the errors introduced by the 2S approximation are completely negligible, especially in terms of energy shifts. RSA is sensitive to the choice of how to truncate the orbital spaces, where other considerations, such as orbital energies instead of number of orbitals, could provide better performance.

Finally, Figure 7 evaluates the absolute errors by LR-TDDFT in estimating RIXS intensities. This is evaluated via the mean ratio between the intensity of the first inelastic peak obtained with LR-TDDFT (optimally aligned with ADC) and the same peak obtained via the 2S ADC approach. Here is where all functionals underperform, generally overestimating the intensity across the board. The actual values of the mean intensity ratios are provided in the SI. Along with the unsurprisingly bad performance of TDHF, LR-TDDFT with the BHandHLYP functional also overestimates the RIXS scattering cross sections by a factor 2–4. For this metric, the previous outliers PBE, M06-L, and SCAN perform quite well, sometimes slightly underestimating the scattering cross sections, as in the case of the N K-edge. We note that PBE was found to underestimate (by a factor ∼2) oscillator strengths of core-excitations,^42^ while BHandHLYP was found to overestimate them by a factor ∼1.5 with respect to ADC.^42^ Range-separated functionals, like rCAM-B3LYP were instead quite close to the ADC reference for XAS.^42^ If the effect of underestimating oscillator strengths and, consequently, transition dipole moments, for core-excitations by PBE is coupled with an overestimation of the core-to-valence transition dipole moments, this could explain the apparent good performance of PBE for the cross-section metric. Similarly, an overestimation of the valence-to-core transition dipole moments across the hybrid and range-separated hybrids functionals coupled to their description of core-excitations would explain the poor performance of BHandHLYP and more reasonable results by the other functionals. Finally, we note that the state-to-state transition density matrices, eq 10, do not include orbital relaxation. In a QR-TDDFT formalism, it was shown that neglecting second order orbital relaxation can lead to a substantial overestimation of excited-state absorption amplitudes.^72^ Depending how orbital relaxation affects the ADC excited-state absorption amplitudes, the omission of this term could play a role in the overestimation of RIXS scattering cross sections by most of the xc functionals. However, we note that second-order orbital relaxation is also missing from the ADC state-to-state transition density matrices used here, so a definitive conclusion on the role of relaxation cannot be drawn from the current benchmark.

**Figure 7:**
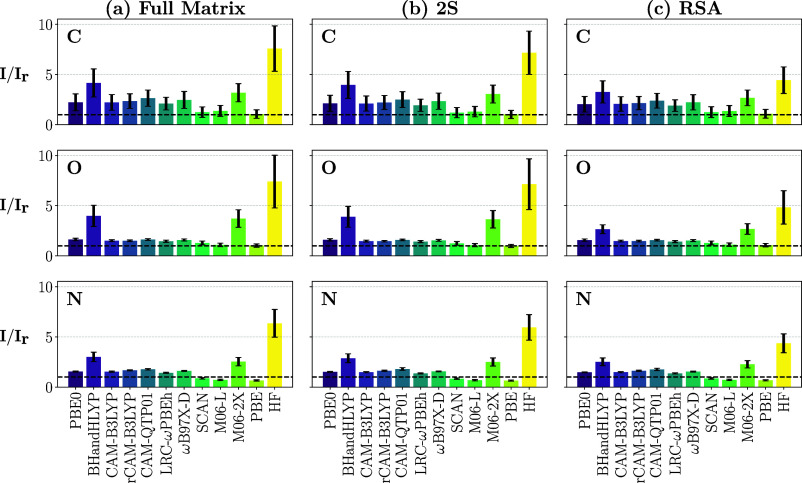
Ratios between RIXS scattering cross sections calculated with 11 different xc functionals and TDHF (I) and the RIXS cross sections calculated with ADC (I_r_). The ratio is reported for the first inelastic peak in the RIXS spectra.

The three statistical metrics discussed above have been evaluated using the LR-TDDFT response matrix, eq 4, however, the conclusions can be extended to TDA as well. In line with similar observations for UV/vis and X-ray absorption calculations,^86^ TDA is found to introduce reasonably small errors in the computation of RIXS, as shown in Table S5 of the Supporting Information. The table presents a comparison of TDA and LR-TDDFT for the N K-edge subset obtained using the 2S approach and the rCAM-B3LYP functional. TDA overestimates RIXS intensities slightly more than LR-TDDFT, but performs similarly in terms of relative spectral features and absolute energy shifts.

To summarize, TDHF and PBE consistently yield the highest relative errors across all metrics, with the exception of intensity ratios where PBE appears to perform well, however this is likely due to a cancellation of errors—a consistent underestimation of the transition dipole moments corresponding to core-excitations combined with an overestimation of the core-to-valence transition dipole moments. The range-separated hybrids perform well across all metrics, with only a small edge dependence. Seeing as the core-excited states enter both the absorption and emission transitions, the RIXS amplitudes depend quadratically on the core-excited eigenvectors. A natural assumption, then, is that an accurate description of the core-excited states is more important than that of the valence. Hence, it could be reasonable to expect that the best-performing functionals of the XABOOM^42^ benchmark would also be the best performing for RIXS. There appears to be some merit to this assumption based on Figures 5 and 6 where CAM-QTP01 (a functional quite similar to the top-performing^42^ CAM100%)^140^ and rCAM-B3LYP (also top-performing for XAS)^42^ are among the best xc functionals across all metrics and edges. However, at the N K-edge in particular, ωB97X-D outperforms both. Notably, ωB97X-D is a functional originally developed—and typically used—for valence-excited states. Taken together, these results suggest that no such universal trend emerges. The position of the features are determined by the ability to describe valence excitations, and so a balanced description should naturally be best. In fact, taking into account the distributions of IADs and the (signed) shifts (see the SI), the range-separated hybrids are very hard to distinguish performance-wise.

In closing, we also note a series of limitations of the current benchmark. Due to the computational cost of our reference method, the benchmark set considered here is composed of small molecules. Although we expect the conclusions to be transferable to medium-sized and large molecules without charge-transfer excitations, this is not necessarily true. Ideally, the benchmark could be extended to larger molecules once more efficient ADC(3) and state-to-state transition density matrix implementations become available, as e.g. in Gator^141^ for ADC(2). Second, the systems investigated here are free of charge-transfer (CT) excitations, where range-separated hybrid functionals outperform regular hybrids. A benchmark set focused on molecules with CT valence-excited states, as e.g. push–pull molecules, could likely show larger performance differences among the hybrid and range-separated hybrid functionals. Finally, we have not considered conformational sampling and nuclear dynamics, therefore our conclusions are not necessarily applicable when modeling vibrational RIXS. In that case, the topology of the core-excited and valence-excited state PES could play an important role and this aspect has not been considered here.

### Large Molecules

The motivation for benchmarking the performance of LR-TDDFT is to be able to apply it in the computation of RIXS spectra for large molecules, where methods like ADC or CC are computationally too expensive. We therefore present an application of the 2S approach to compute the RIXS spectrum of C_60_, targeting the lowest-energy core resonance, 1s → t_1*u*_. The calculation, performed using the PBE0 functional in combination with the def2-SVPD basis set, is shown in Figure 8 in comparison to an experimental spectrum from ref 61.

**Figure 8:**
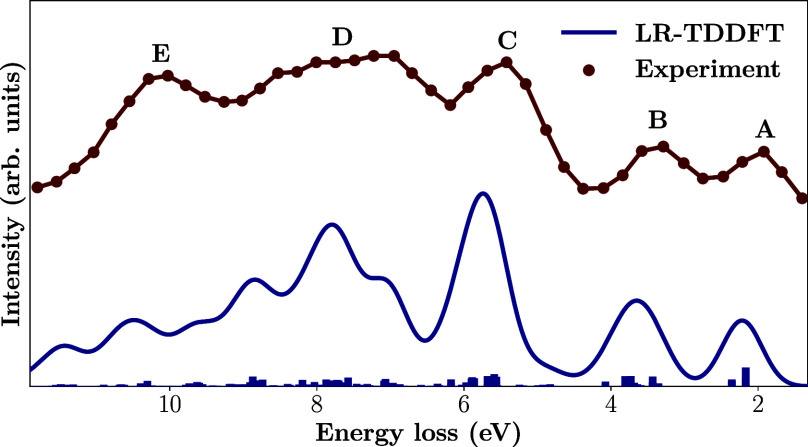
Calculated RIXS spectrum of fully symmetric C_60_ at the 1s → t_1*u*_ resonance with θ = π/2 obtained via 2S LR-TDDFT with the PBE0 functional and def2-SVPD basis set using C 1s core orbitals localized by the Boys procedure. A Gaussian broadening of 0.6 eV fwhm has been applied to the calculated discrete spectrum. Experimental data extracted from ref 61 with WebPlotDigitzer.^142^

As seen in Figure 8, most of the features of the experiment are well reproduced, with only a small shift toward higher energies. From a symmetry point of view, the **B** feature should be forbidden, as should be large parts of **C** and **D**, and their presence in the experiment is mostly due to vibronic coupling,^61^ where dynamical symmetry breaking results in the localization of the core-hole. This core-excited Jahn–Teller effect which takes place in the resonant case can be modeled using localized core orbitals,^95,143^ the approach adopted here. The intensity of feature **E** at around 10.27 eV energy loss is not well reproduced by the calculation, possibly explained by the basis set choice which may not be large and diffuse enough to capture this high energy region. Additionally, the incident photon beam has a finite line-width in the experiment, while a narrow line-width δ­(ω) is used in our implementation. Explicit vibrational broadening and vibrational fine-structure is naturally also not present in the computational model. From a vertical-excitation point of view the agreement is very good.

## Conclusions

In conclusion, we have implemented the 2S and RSA approximations for RIXS at the LR-TDDFT theory level in the VeloxChem program and benchmarked the performance of different xc functionals in comparison to 2S ADC, using ADC(3) to describe valence-excited states and CVS-ADC(2)-x for core-excitations. The performance of LR-TDDFT was assessed both in terms of *relative* peak positions and intensities, as well as absolute energies and scattering cross sections. Most hybrid and all range-separated hybrid functionals performed well across all metrics, noting a small overestimation of the RIXS scattering cross sections, but with a good description of relative spectral features and fairly small energy shifts. Since the analysis is performed in energy loss, the errors in core-excitation energies cancel out with errors in the core-to-valence transition energies. The energy errors, therefore, appear to follow the trends observed for the xc functionals in predicting valence-excitation energies. LR-TDDFT can be efficiently applied to compute electronic RIXS spectra of large molecules, but conformational sampling should be considered to be able to fully capture the experimental spectral features.

## Supplementary Material



## Notes

Comparisons between localization schemes; comparisons of two-shot (2S) ADC results calculated with different basis sets; parameters of the DFT functionals used in the benchmark; distributions of energy shifts, integrated absolute differences (IADs), and tables of the mean intensity ratios between the first inelastic peak determined by LR-TDDFT and the first inelastic peak determined by 2S ADC; effects of the Tamm-Dancoff approximation; and comparisons between 2S LR-TDDFT and the restricted subspace approximation (RSA) (PDF)
