# Supplementary material for: Resonant Inelastic X‑ray Scattering: How Well Does LR-TDDFT Perform?
Source: J Phys Chem A. 2025 Sep 17;129(38):8783–97. doi: 10.1021/acs.jpca.5c04528 (PMC12478873; doi:10.1021/acs.jpca.5c04528)
Supplement: Supplementary file 1 [file jp5c04528_si_001.pdf]

Supporting Information — Resonant Inelastic X-ray Scattering: How well does LR-TDDFT perform?

Erik Vitols,*,†,‡ Vinícius Vaz da Cruz,¶ Thomas Fransson,§ and Iulia Emilia Brumboiu*,†

†*Faculty of Physics, Astronomy and Informatics, Nicolaus Copernicus University in Toruń, 87-100 Toruń, Poland*

‡*Division of Theoretical Chemistry and Biology, KTH Royal Institute of Technology, 100 44 Stockholm, Sweden*

¶*Helmholtz-Zentrum Berlin für Materialien und Energie, Institute for Methods and Instrumentation for Synchrotron Radiation Research, Albert-Einstein-Strasse 15, D-12489 Berlin, Germany*

§*No affiliation*

E-mail: evitols@kth.se; iubr@umk.pl

Included here are: **(1)** comparisons between localization schemes; **(2)** comparisons of two-shot (2S) ADC results calculated with different basis sets; **(3)** parameters of the DFT functionals used in the benchmark; **(4)** the distributions of energy shifts, integrated absolute differences (IADs), and tables of the mean intensity ratios between the first inelastic peak determined by LR-TDDFT and the first inelastic peak determined by 2S ADC; **(5)** effects of the Tamm-Dancoff approximation; **(6)** comparisons between 2S LR-TDDFT and the restricted subspace approximation (RSA).

Localization of the core orbitals

Figure S1 shows a comparison between the RIXS spectrum of ethylene calculated using LR-TDDFT within the 2S approach, with the PBE0 functional,[1] aug-cc-pVTZ[2] basis set, and two approaches to localize the core orbitals. The shaded gray spectrum is calculated for a perfectly symmetric ethylene, where the C 1s orbitals are localized using Boys localization.[3] The spectrum in red is obtained by slightly distorting the molecular geometry to localize the core orbitals. The error introduced by distorting the molecule is reasonably small, both in terms of energy position and intensities of the RIXS peaks.

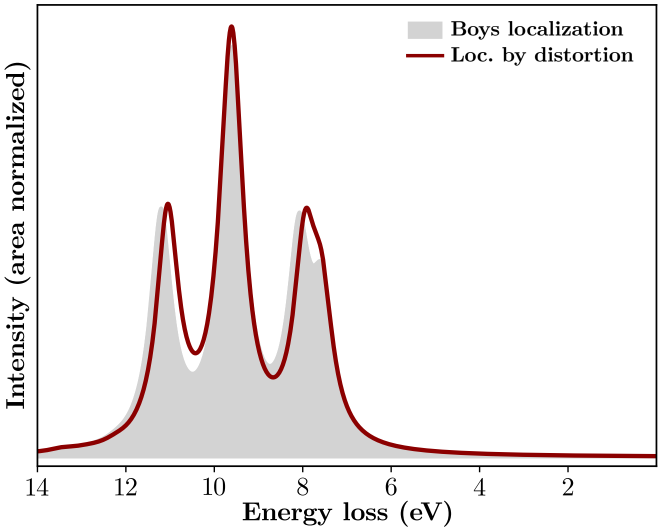

Figure S1: Comparison between the calculated RIXS spectra of the ethylene molecule calculated using two different approaches to localize the core orbitals.

Basis Set Dependence

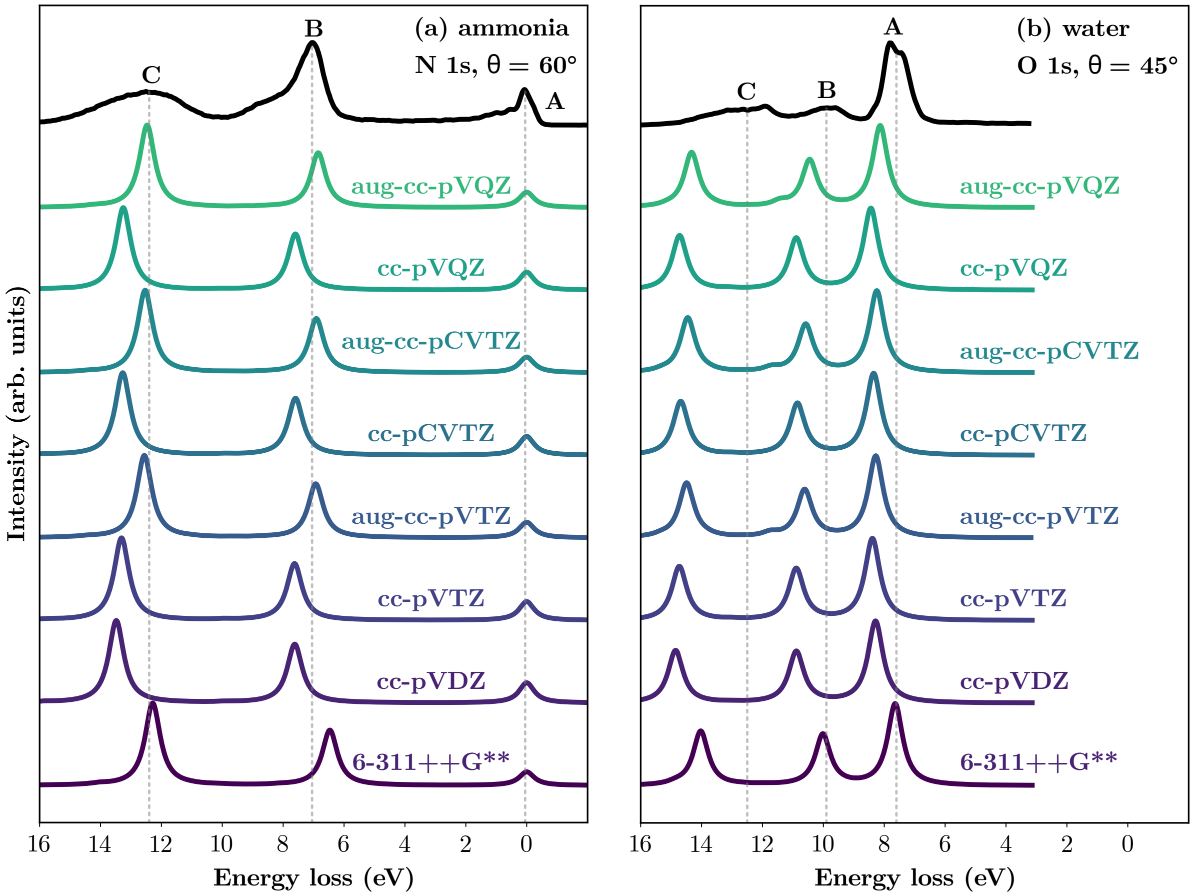

Figure S2: Comparison between RIXS spectra calculated using 2S ADC(3):CVS-ADC(2)-x with different basis sets and experimental RIXS spectra of (a) aqueous ammonia at the N K-edge[4] and (b) gas phase water at the O K-edge.[5] The incident photon energy corresponds to the lowest core-excitation and θ denotes the angle between the incident photon polarization and the scattered photon direction. No energy shift of the calculated data has been performed.

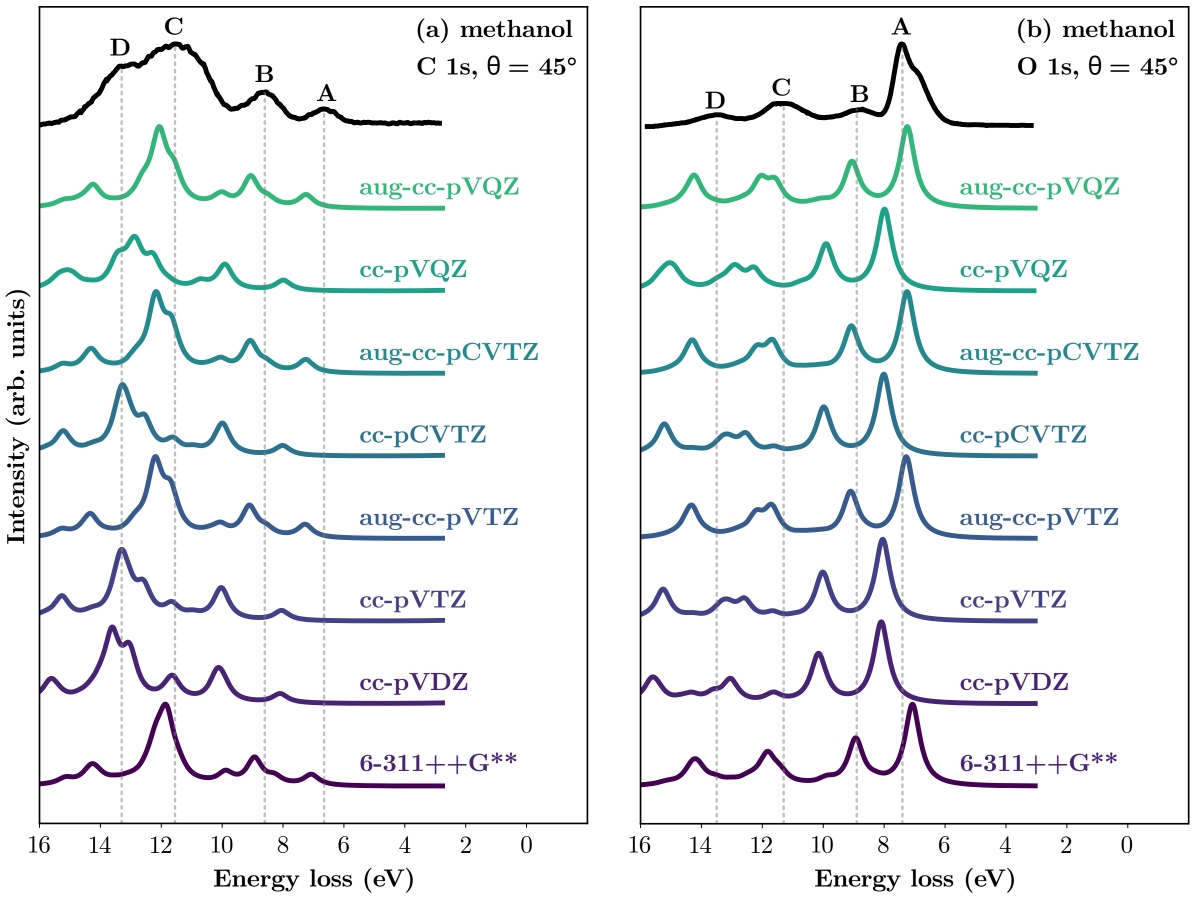

Figure S3: Comparison between RIXS spectra calculated using 2S ADC(3):CVS-ADC(2)-x with different basis sets and experimental RIXS spectra of (a) gas methanol at the C and (b) O K-edges.[6] The incident photon energy corresponds to the lowest core-excitation and θ denotes the angle between the incident photon polarization and the scattered photon direction. No energy shift of the calculated data has been performed.

Exchange and correlation functionals

The details of the exchange and correlation functionals included in the benchmark are listed, alongside their functional forms, parameters, and the grid size used for numerical integration, in Table S1. For range-separated functionals, short and long range exchange contribution are indicated by the superscripts SR and LR, respectively, while the range-separation parameter is denoted by μ or ω, depending on the functional. The evaluation of exchange-correlation functionals is carried out through the `Libxc` library.[7] The grid level, given in the last column of the table, is the default value used in `VeloxChem`[8] for different functionals to ensure

Table S1: The functional forms and related parameters of the exchange and correlation functionals included in the benchmark. The last column shows the default grid level used in VeloxChem for numerical integration.

Functional	Functional Form	Parameters	Grid
PBE[9]	$E_{\mathrm{x}}^{\mathrm{PBE}} + E_{\mathrm{c}}^{\mathrm{PBE}}$	–	4
PBE0[1]	$a_{\mathrm{x}} E_{\mathrm{HF}} + b_{\mathrm{x}} E_{\mathrm{x}}^{\mathrm{PBE}} + E_{\mathrm{c}}^{\mathrm{PBE}}$	$a_{\mathrm{x}} = 0.25$, $b_{\mathrm{x}} = 0.75$	4
BHandHLYP[10]	$a_{\mathrm{x}} E_{\mathrm{x}}^{\mathrm{HF}} + b_{\mathrm{x}} E_{\mathrm{x}}^{\mathrm{B88}} + E_{\mathrm{c}}^{\mathrm{LYP}}$	$a_{\mathrm{x}} = 0.50$, $b_{\mathrm{x}} = 0.50$	4
CAM-B3LYP[11]	$a_{\mathrm{x}}^{\mathrm{SR}} E_{\mathrm{x,SR}}^{\mathrm{HF}} + b_{\mathrm{x}}^{\mathrm{SR}} E_{\mathrm{x,SR}}^{\mathrm{B88}} + a_{\mathrm{x}}^{\mathrm{LR}} E_{\mathrm{x,LR}}^{\mathrm{HF}}$ $+ b_{\mathrm{x}}^{\mathrm{LR}} E_{\mathrm{x,LR}}^{\mathrm{B88}} + a_{\mathrm{c}} E_{\mathrm{c}}^{\mathrm{LYP}} + b_{\mathrm{c}} E_{\mathrm{c}}^{\mathrm{VWN}}$	$a_{\mathrm{x}}^{\mathrm{SR}} = 0.19$, $b_{\mathrm{x}}^{\mathrm{SR}} = 0.46$, $a_{\mathrm{x}}^{\mathrm{LR}} = 0.65$, $b_{\mathrm{x}}^{\mathrm{LR}} = 0.35$, $a_{\mathrm{c}} = 0.81$, $b_{\mathrm{c}} = 0.19$, $\mu = 0.33$ bohr^{-1}	4
rCAM-B3LYP[12]	$a_{\mathrm{x}}^{\mathrm{SR}} E_{\mathrm{x,SR}}^{\mathrm{HF}} + b_{\mathrm{x}}^{\mathrm{SR}} E_{\mathrm{x,SR}}^{\mathrm{B88}} + c_{\mathrm{x}} E_{\mathrm{x}}^{\mathrm{Slater}}$ $+ a_{\mathrm{x}}^{\mathrm{LR}} E_{\mathrm{x,LR}}^{\mathrm{HF}} + b_{\mathrm{x}}^{\mathrm{LR}} E_{\mathrm{x,LR}}^{\mathrm{B88}} + a_c E_{\mathrm{c}}^{\mathrm{LYP}}$	$a_{\mathrm{x}}^{\mathrm{SR}} = 0.1835$, $b_{\mathrm{x}}^{\mathrm{SR}} = 0.9498$, $a_{\mathrm{x}}^{\mathrm{LR}} = 1.1333$, $b_{\mathrm{x}}^{\mathrm{LR}} = 0.0026$, $c_{\mathrm{x}} = -0.1359$, $a_{\mathrm{c}} = 1.0$, $\mu = 0.33$ bohr^{-1}	4
CAM-QTP01[13]	$a_{\mathrm{x}}^{\mathrm{SR}} E_{\mathrm{x,SR}}^{\mathrm{HF}} + b_{\mathrm{x}}^{\mathrm{SR}} E_{\mathrm{x,SR}}^{\mathrm{B88}} + a_{\mathrm{x}}^{\mathrm{LR}} E_{\mathrm{x,LR}}^{\mathrm{HF}} +$ $b_{\mathrm{x}}^{\mathrm{LR}} E_{\mathrm{x,LR}}^{\mathrm{B88}} + a_{\mathrm{c}}^{\mathrm{LYP}} + b_{\mathrm{c}}^{\mathrm{VWN}}$	$a_{\mathrm{x}}^{\mathrm{SR}} = 0.33$, $b_{\mathrm{x}}^{\mathrm{SR}} = 0.77$, $a_{\mathrm{x}}^{\mathrm{LR}} = 1.0$, $b_{\mathrm{x}}^{\mathrm{LR}} = 0.0$, $\mu = 0.31$ bohr^{-1}	4
LRC-ωPBEh[14]	$a_{\mathrm{x}} E_{\mathrm{x,SR}}^{\mathrm{HF}} + b_{\mathrm{x}} E_{\mathrm{x,SR}}^{\mathrm{PBE}}$ $+ E_{\mathrm{x,LR}}^{\mathrm{HF}} + E_{\mathrm{c}}^{\mathrm{PBE}}$	$a_{\mathrm{x}} = 0.20$, $b_{\mathrm{x}} = 0.80$, $\omega = 0.20$ bohr^{-1}	4
ωB97X-D[15]	$a_{\mathrm{x}} E_{\mathrm{x,\,SR}}^{\mathrm{HF}} + b_{\mathrm{x}} E_{\mathrm{x,SR}}^{\mathrm{B97}}$ $+ E_{\mathrm{x,\,LR}}^{\mathrm{HF}} + E_{\mathrm{c}}^{\mathrm{B97}}$	$a_{\mathrm{x}} = 0.22036$, $b_{\mathrm{x}} = 0.77964$ $\omega = 0.20$ bohr^{-1}	4
M06-L[16]	$E_{\mathrm{x}}^{\mathrm{M06\text{-}L}} + E_{\mathrm{c}}^{\mathrm{M06\text{-}L}}$	–	6
M06-2X[17]	$a_{\mathrm{x}} E_{\mathrm{x}}^{\mathrm{HF}} + b_{\mathrm{x}} E_{\mathrm{x}}^{\mathrm{M06\text{-}2X}} + E_{\mathrm{c}}^{\mathrm{M06\text{-}2X}}$	$a_{\mathrm{x}} = 0.54, b_{\mathrm{x}} = 0.44$	6
SCAN[18]	$E_{\mathrm{x}}^{\mathrm{SCAN}} + E_{\mathrm{c}}^{\mathrm{SCAN}}$	–	7

convergence of the ground state total energy with respect to the grid size. The grid level determines the size of the grid, where a grid level 1 is the smallest least accurate grid, while grid level 8 is the largest and most accurate one. For reference, a grid level 1 generates a grid with 30 radial points and 110 angular points in the case of a C, N, or O atom, while a grid level 8 generates a grid with 200 radial and 2030 angular points for the same atom types.

Benchmark distributions

The distributions of energy shifts and optimally shifted IADs are given in the following. These distributions were used to determine the statistical metrics shown in the main text. Note that the shifts given here are the *signed* shifts and so the mean of these distributions will not agree with those of the mean *absolute* shifts reported in the main text. A Gaussian distribution was used to model the energy shifts. The Gaussian/normal distribution has probability density function

$$f(x; \mu, \sigma^2) = \frac{1}{\sqrt{2\pi\sigma^2}} e^{-\frac{(x-\mu)^2}{2\sigma^2}}, \tag{1}$$

with μ the mean, and σ^2 the variance. Given that the IAD is strictly positive and exhibits an asymmetric distribution, a Gamma distribution is instead used to model it. The Gamma distribution has probability density function

$$f(x; \alpha, \theta) = \frac{x^{\alpha-1} e^{-x/\theta}}{\theta^\alpha \Gamma(\alpha)} \text{ for } x > 0, \text{ with } \alpha, \theta > 0, \tag{2}$$

where $\Gamma(\alpha)$ is the gamma function. The mean is given by: $\mu = \alpha\theta$, and the variance by: $\sigma^2 = \alpha\theta^2$.

Distributions of Energy Shifts

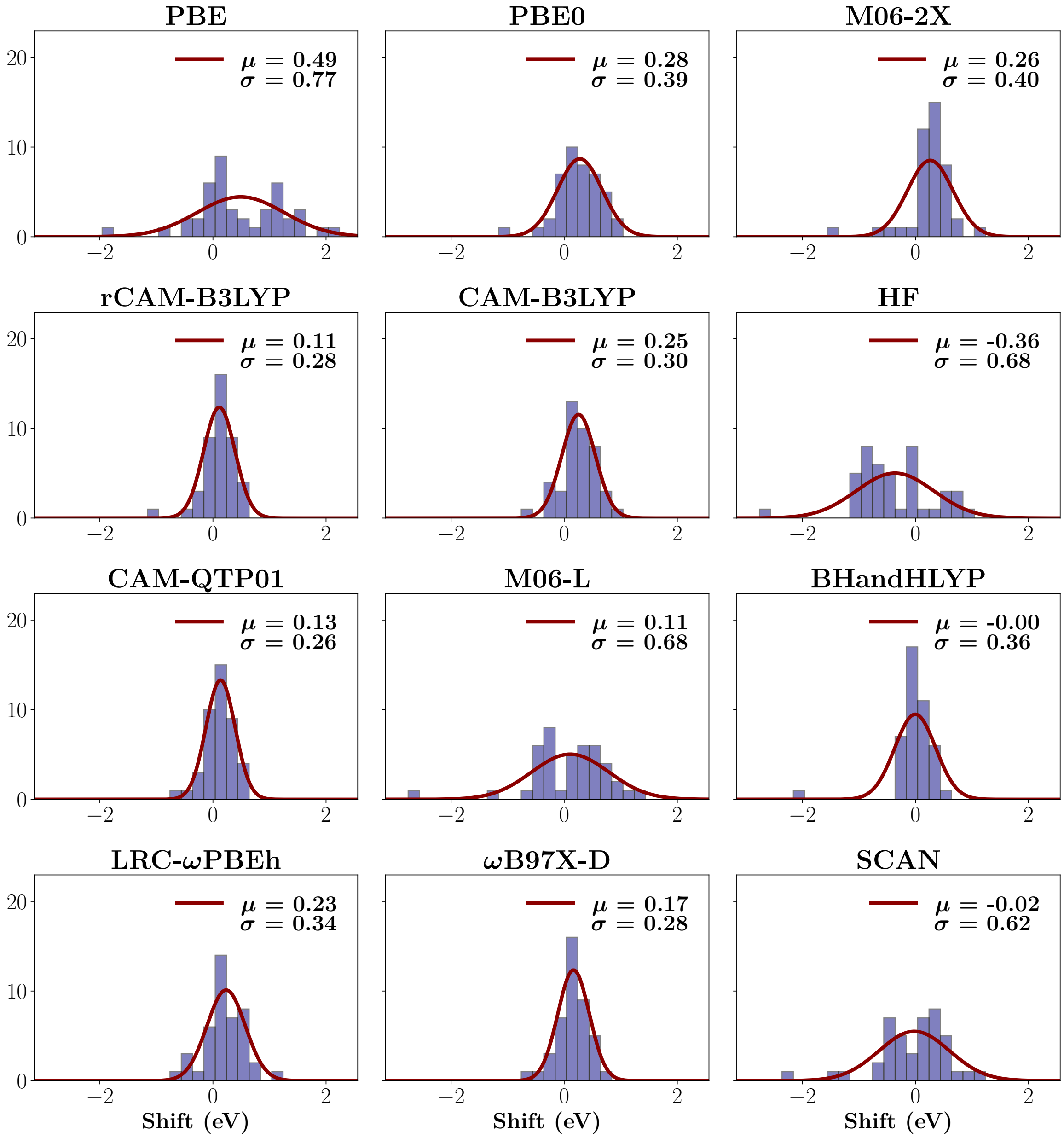

Figure S4: Energy shifts required to optimally shift the spectra excited through the **C 1s** resonance, in the **full-matrix diagonalization** approach.

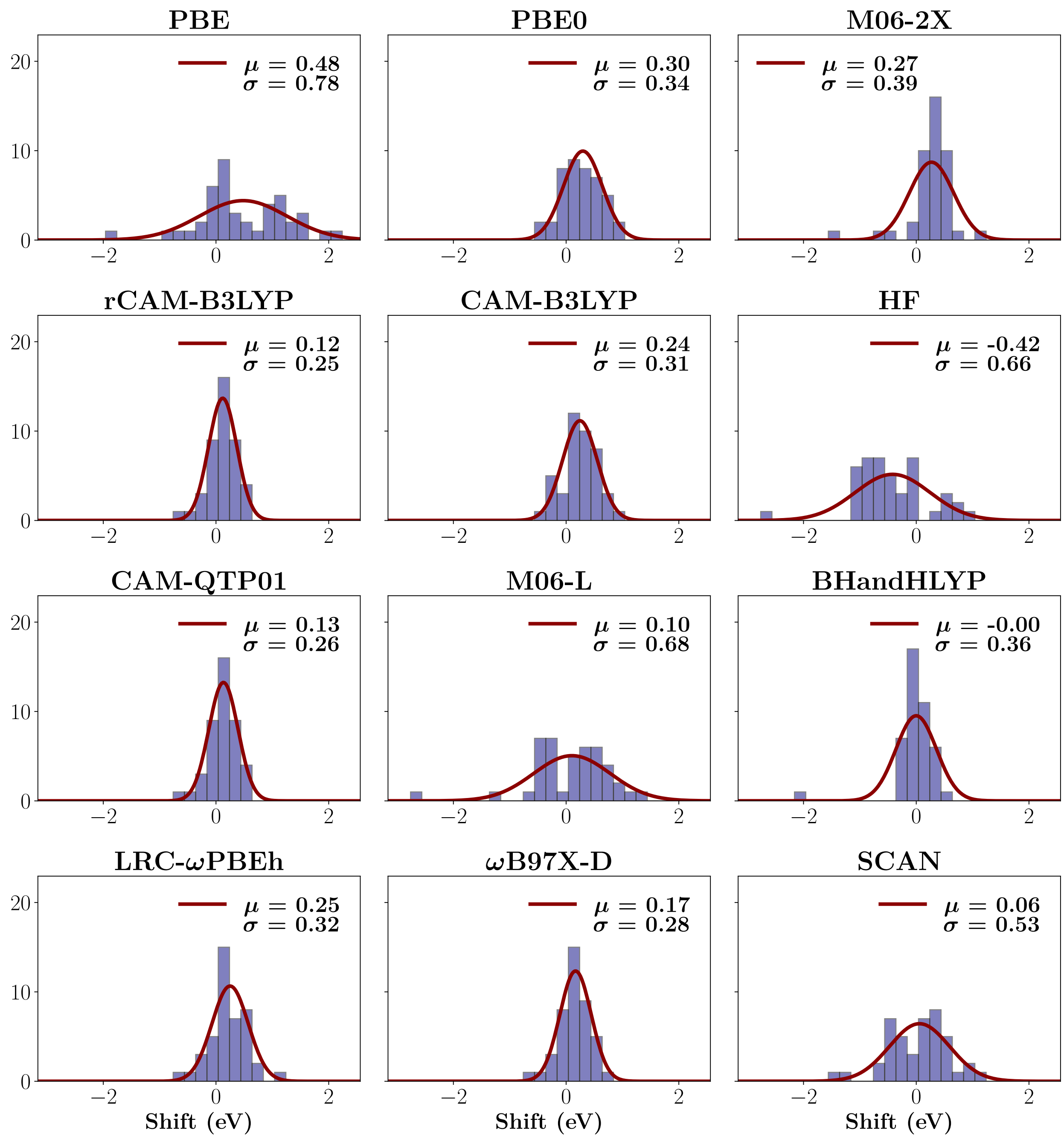

Figure S5: Energy shifts required to optimally shift the spectra excited through the **C 1s** resonance, in the **2S** approach.

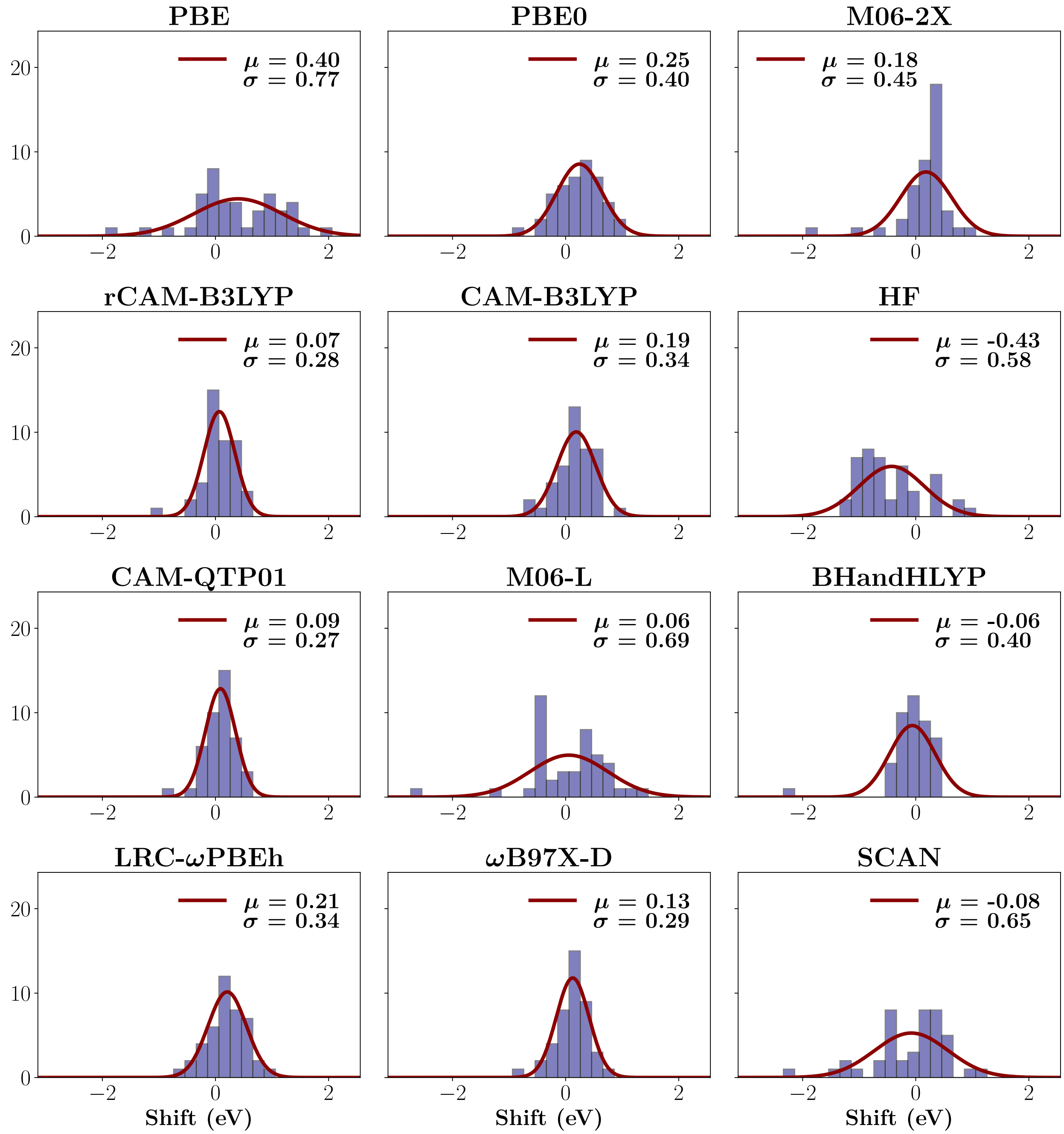

Figure S6: Energy shifts required to optimally shift the spectra excited through the **C 1s** resonance, in the **RSA** approach.

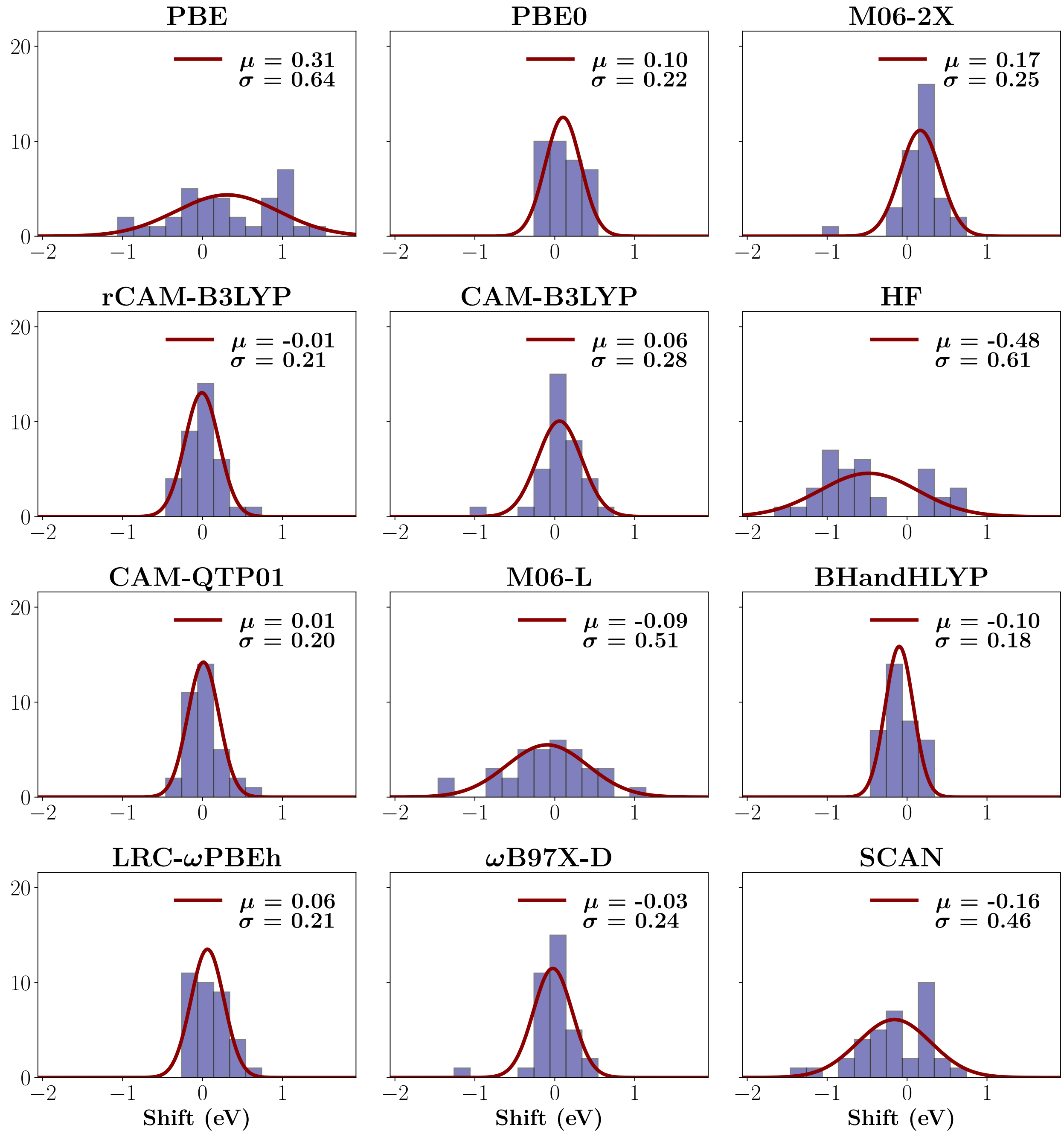

Figure S7: Energy shifts required to optimally shift the spectra excited through the **N 1s** resonance, in the **full-matrix diagonalization** approach.

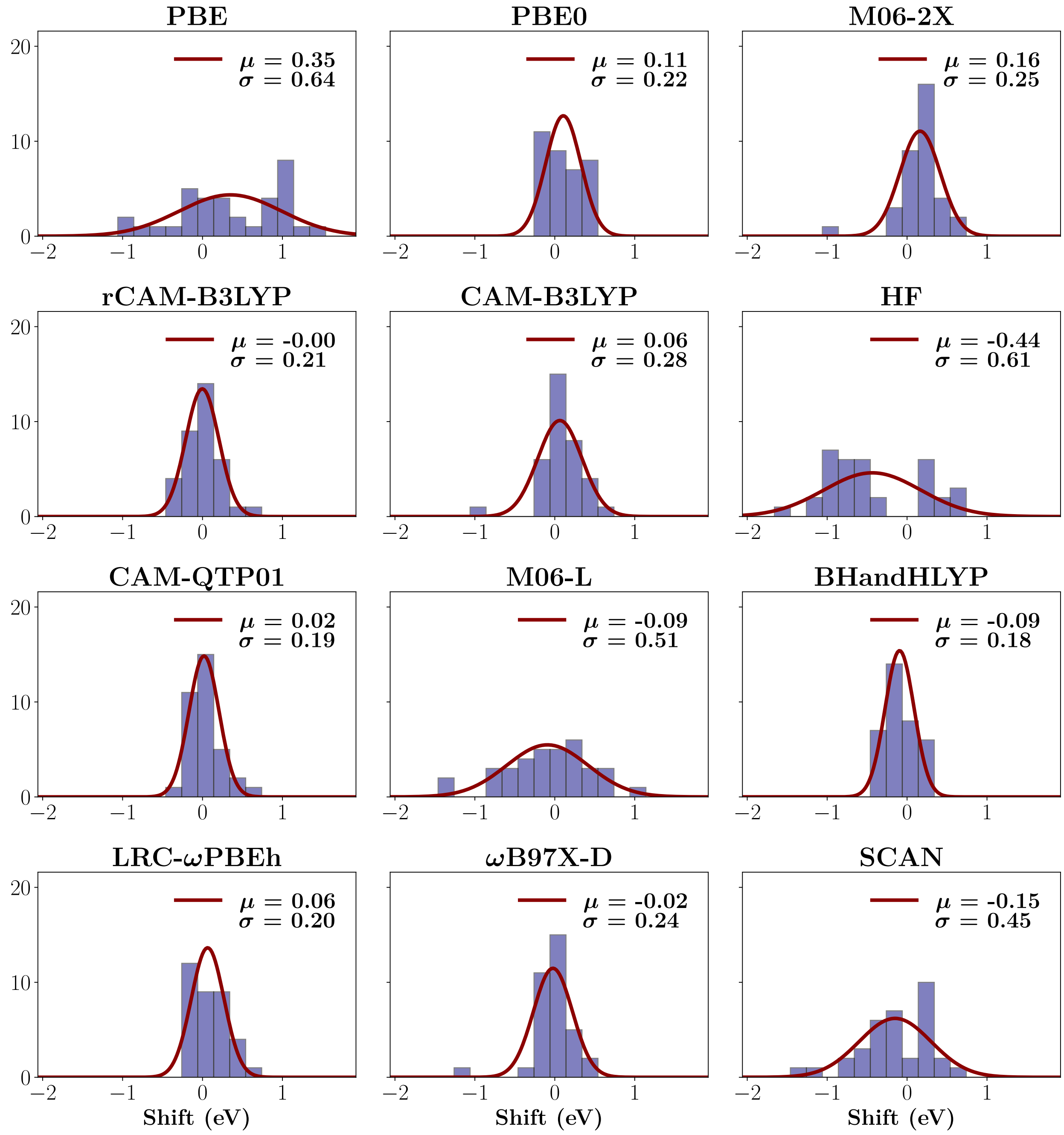

Figure S8: Energy shifts required to optimally shift the spectra excited through the **N 1s** resonance, in the **2S** approach.

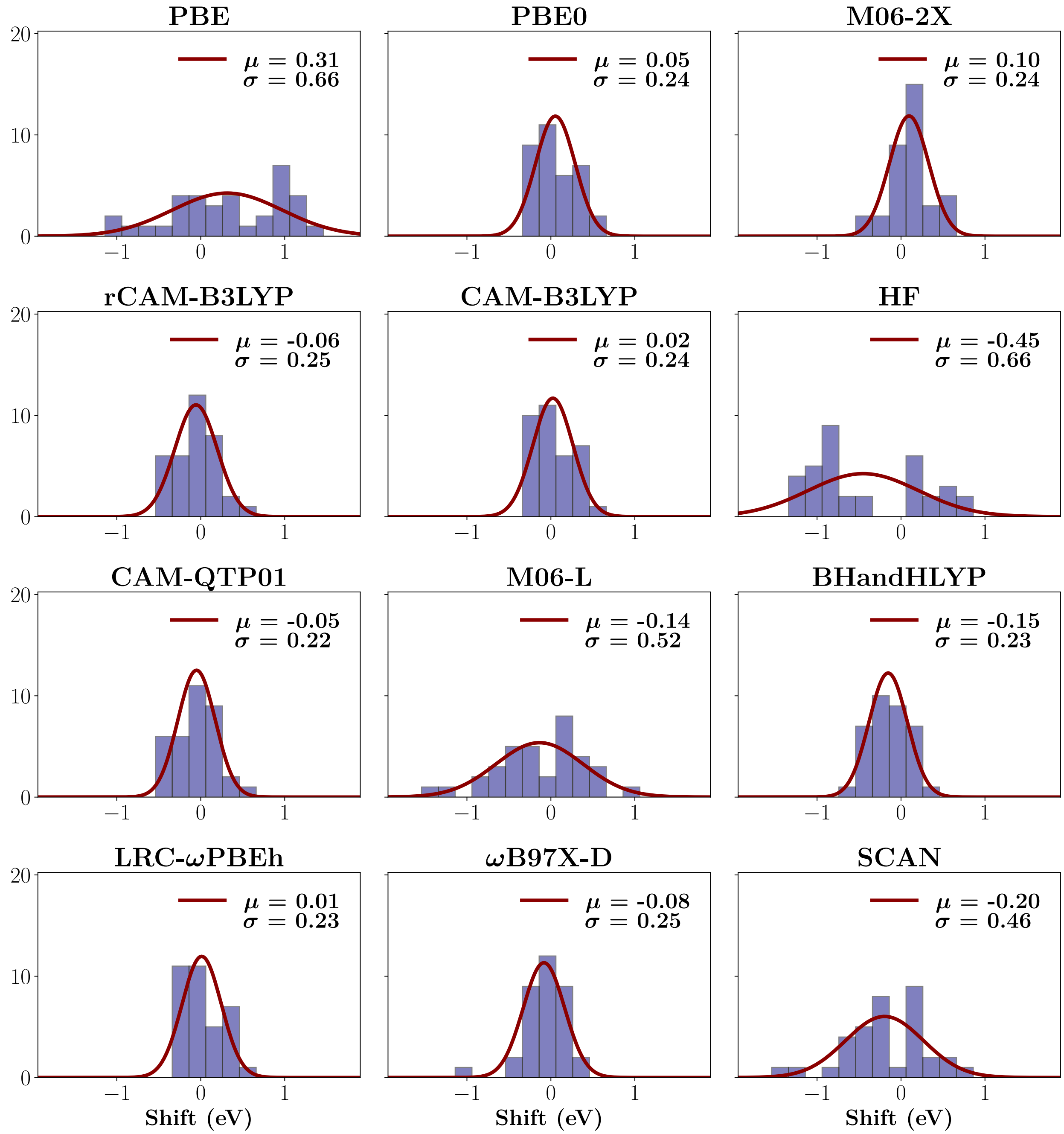

Figure S9: Energy shifts required to optimally shift the spectra excited through the **N 1s** resonance, in the **RSA** approach.

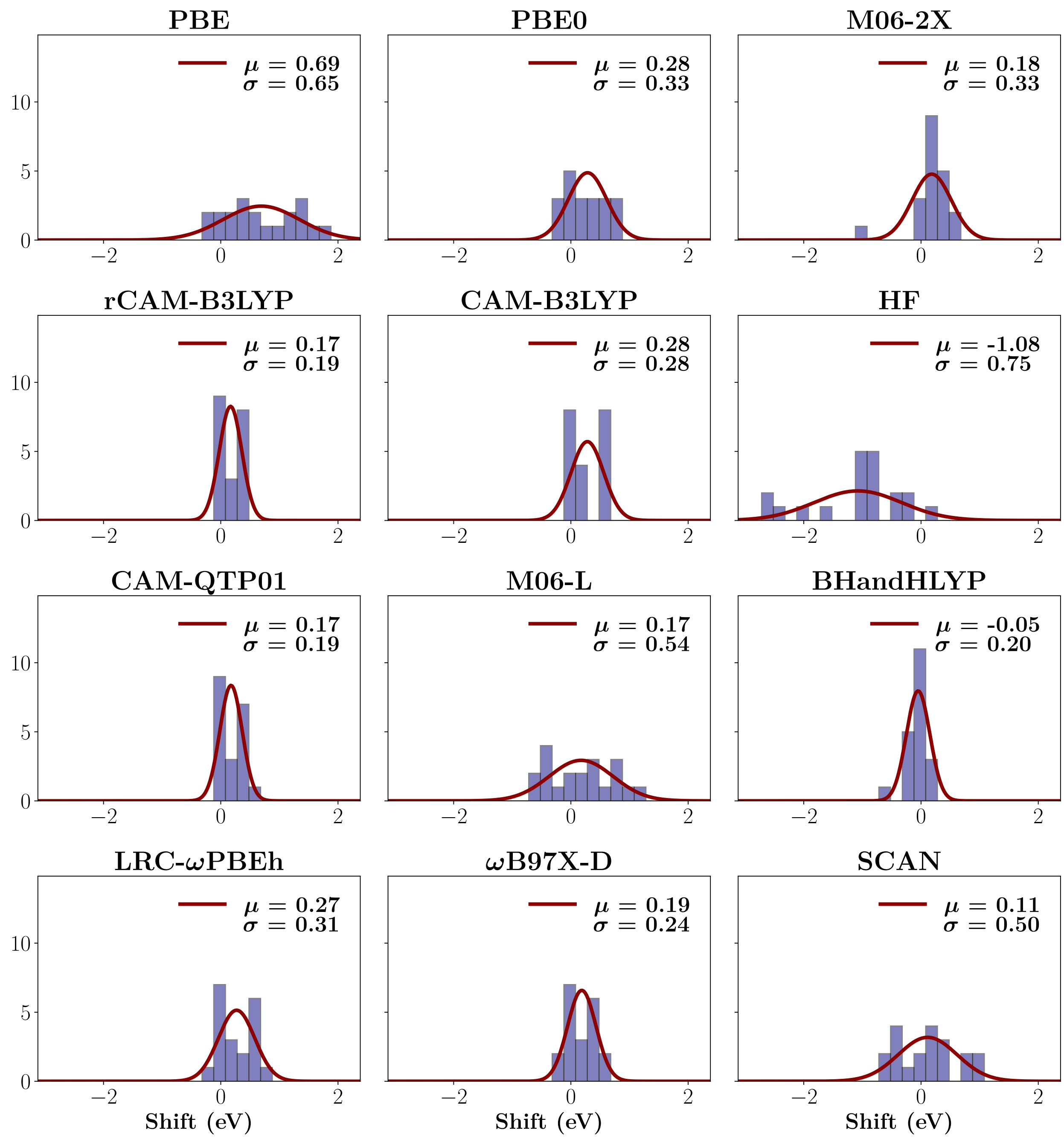

Figure S10: Energy shifts required to optimally shift the spectra excited through the **O 1s** resonance, in the **full-matrix diagonalization** approach.

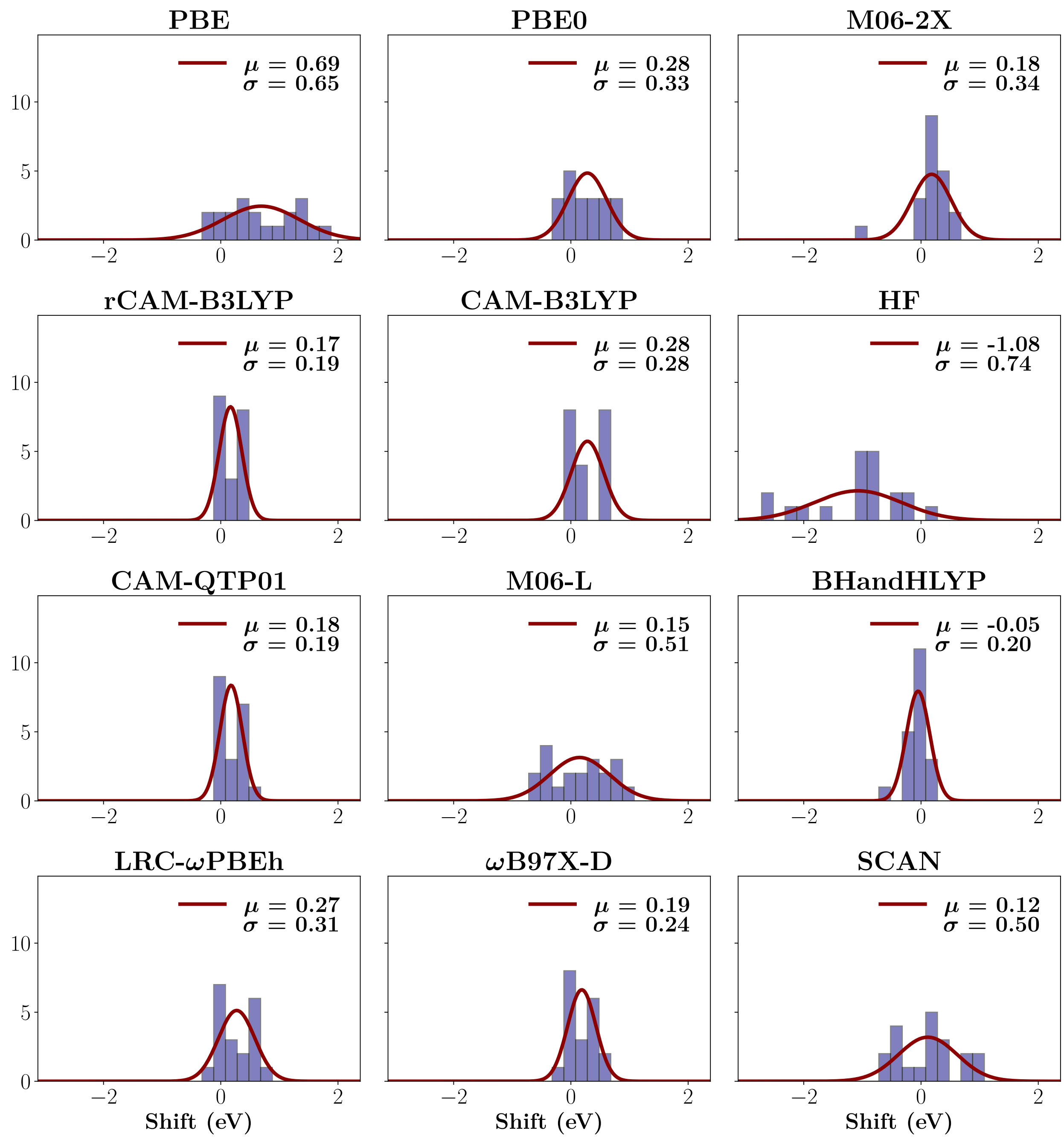

Figure S11: Energy shifts required to optimally shift the spectra excited through the **O 1s** resonance, in the **2S** approach.

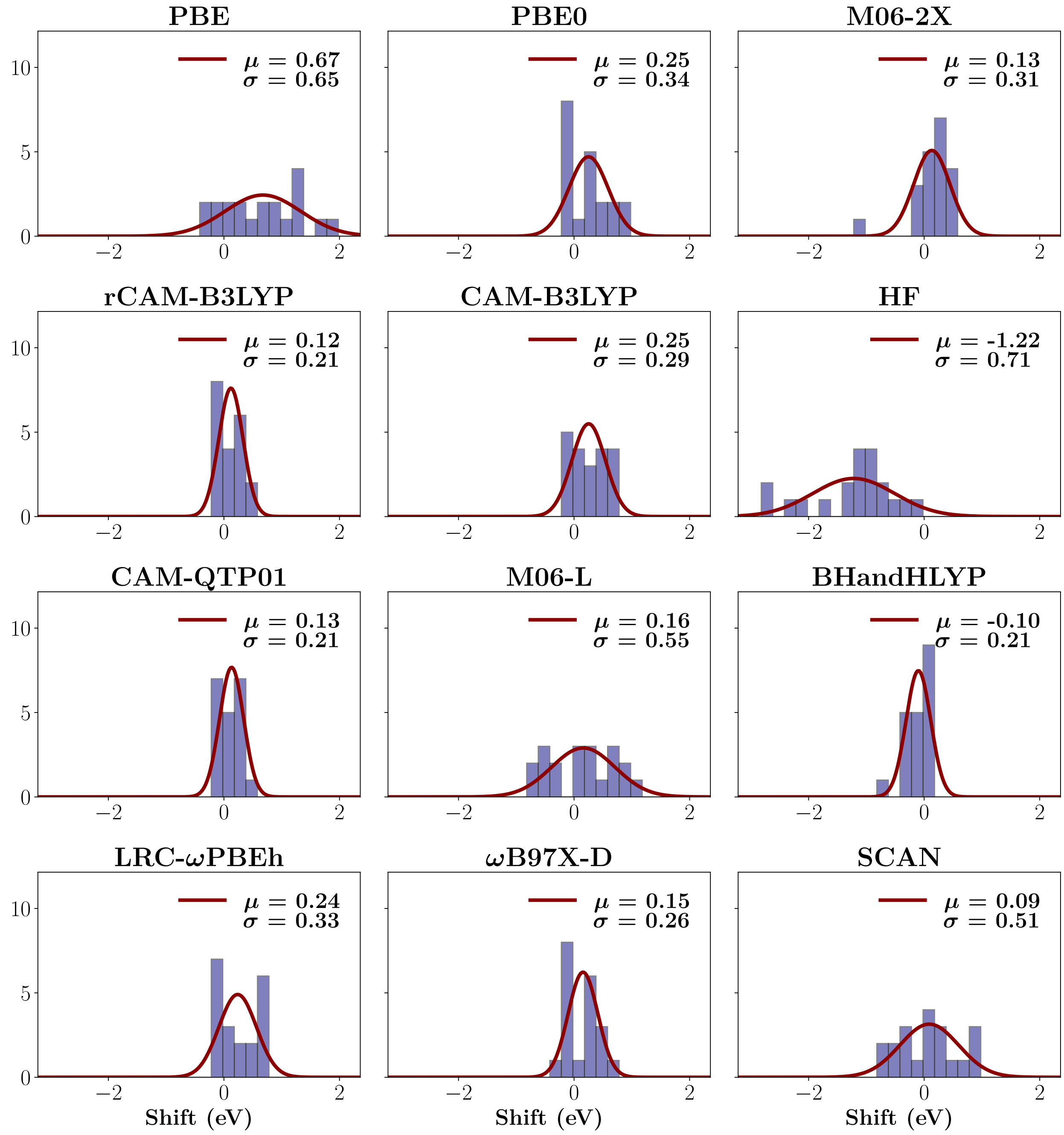

Figure S12: Energy shifts required to optimally shift the spectra excited through the **O 1s** resonance, in the **RSA** approach.

Distributions of IADs

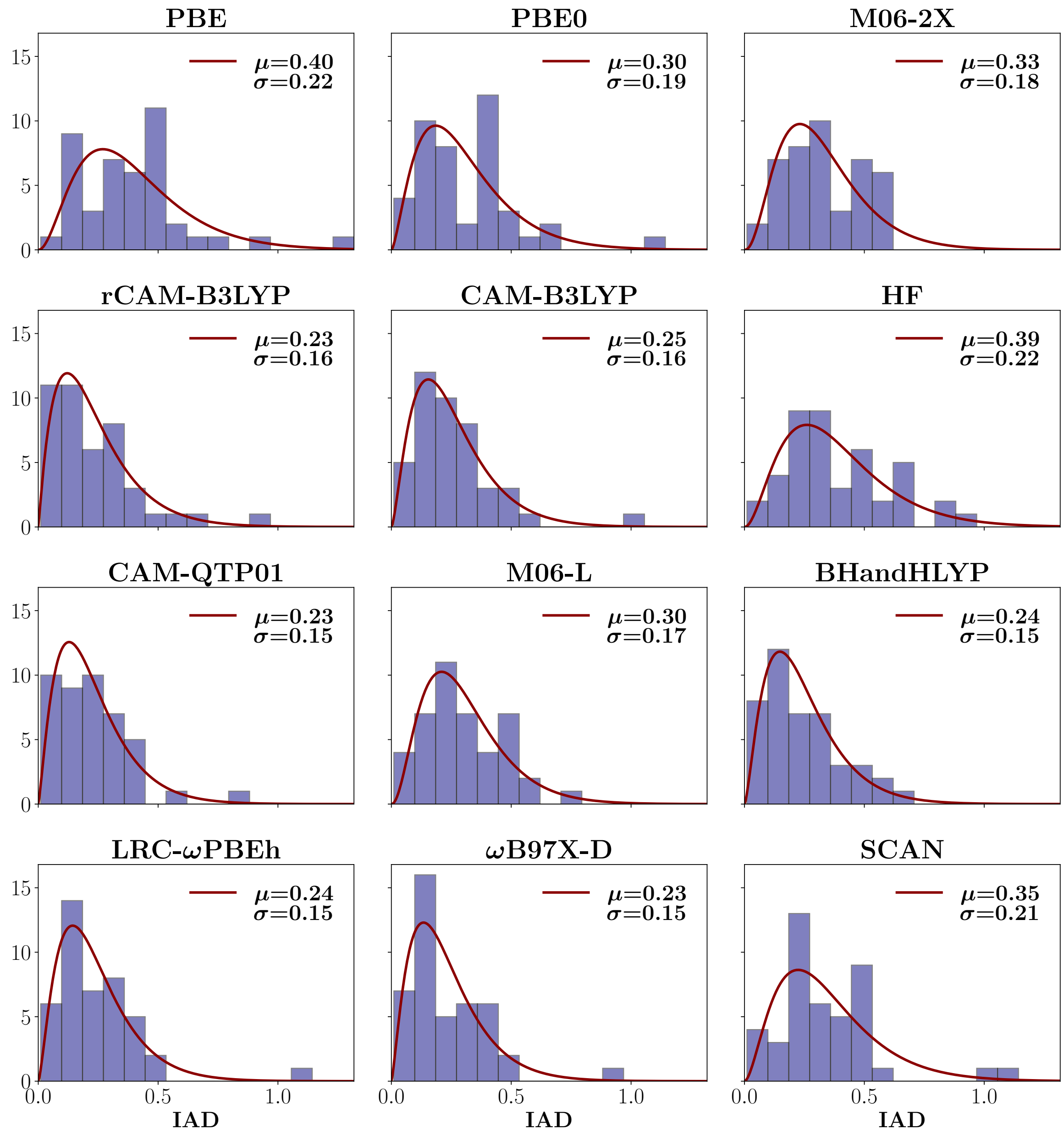

Figure S13: The distribution of optimally shifted IADs, on the **C 1s** resonance, in the **full-matrix diagonalization** approach.

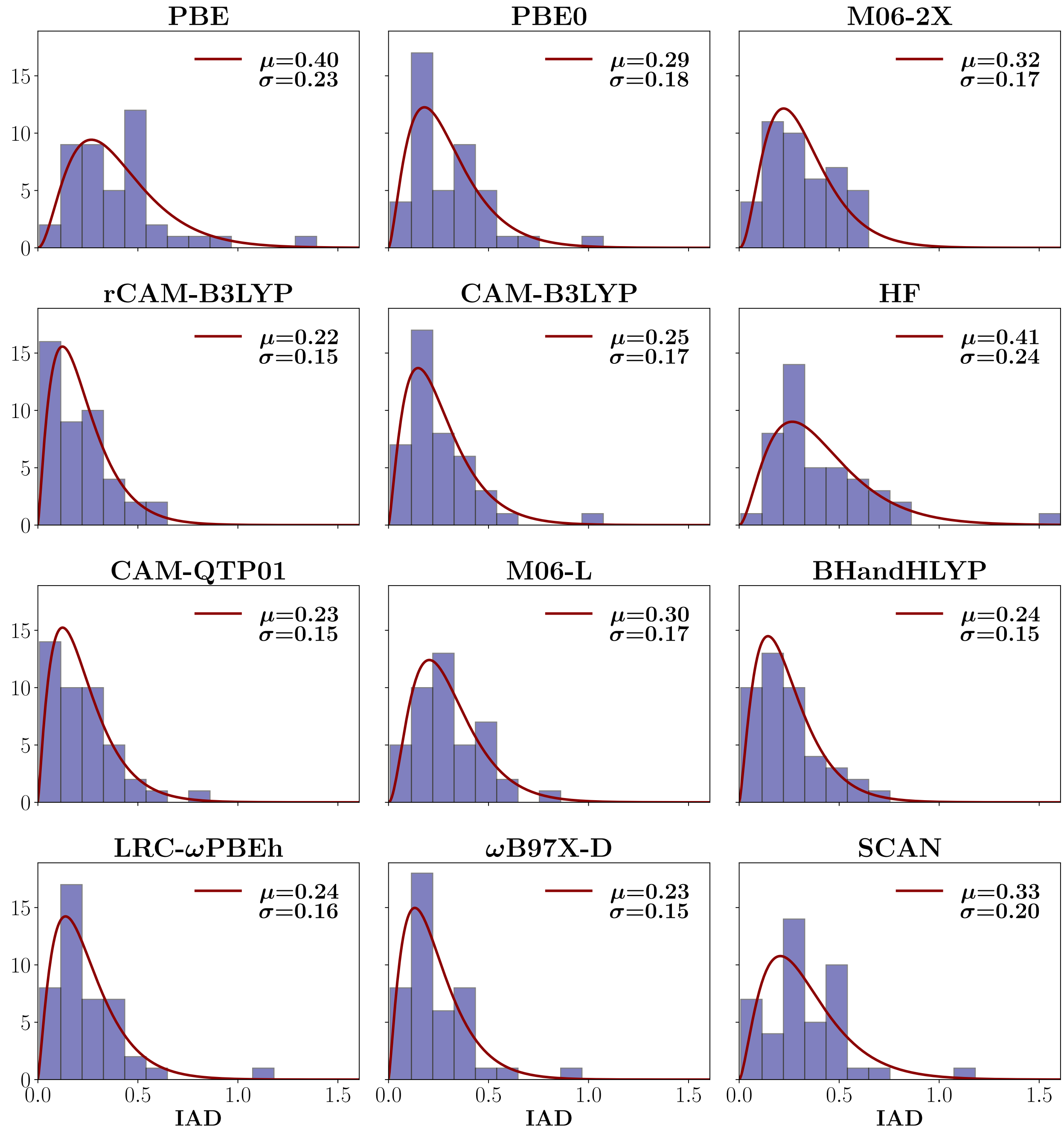

Figure S14: The distribution of optimally shifted IADs, on the **C 1s** resonance, in the **2S** approach.

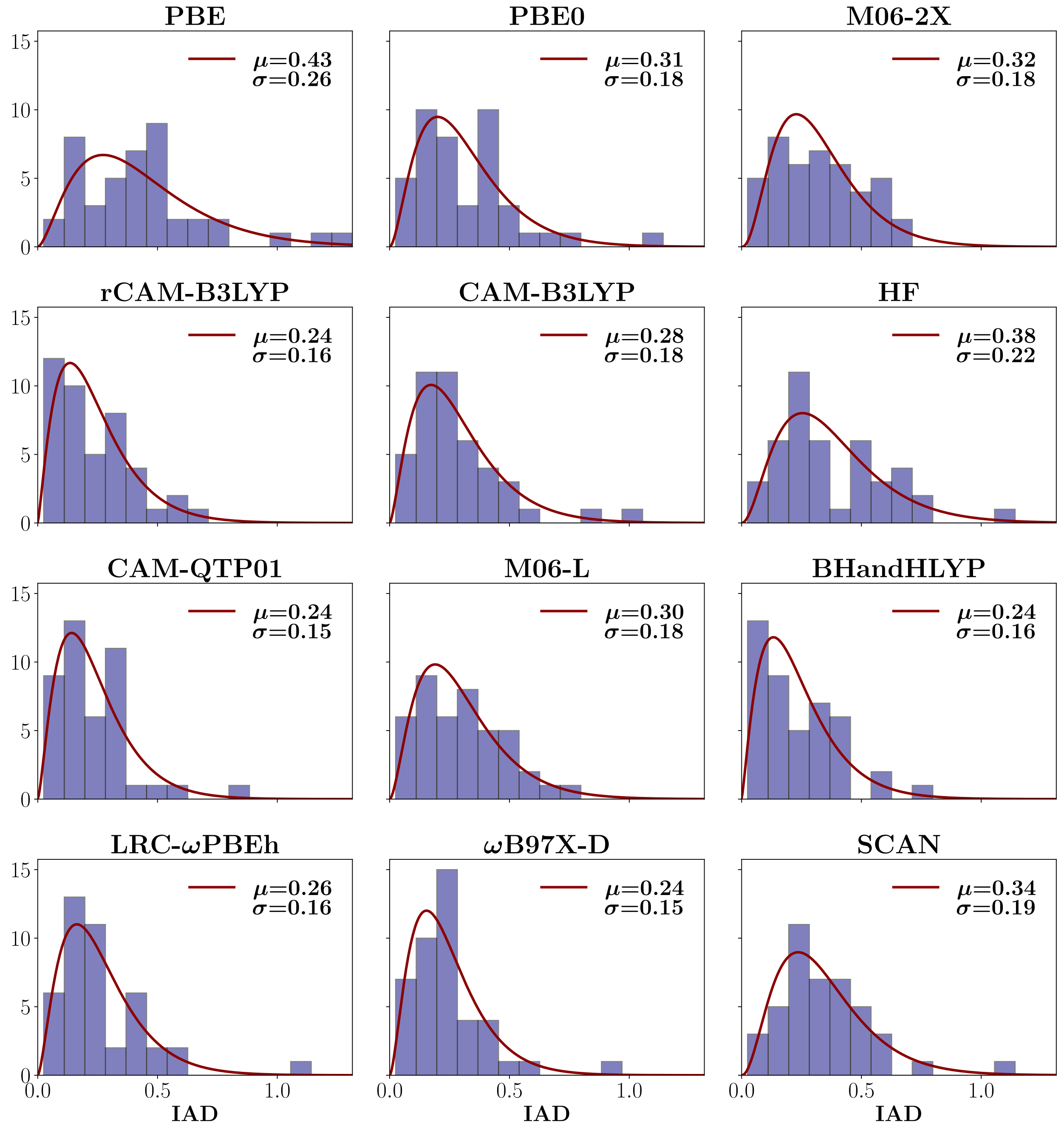

Figure S15: The distribution of optimally shifted IADs, on the **C 1s** resonance, in the **RSA** approach.

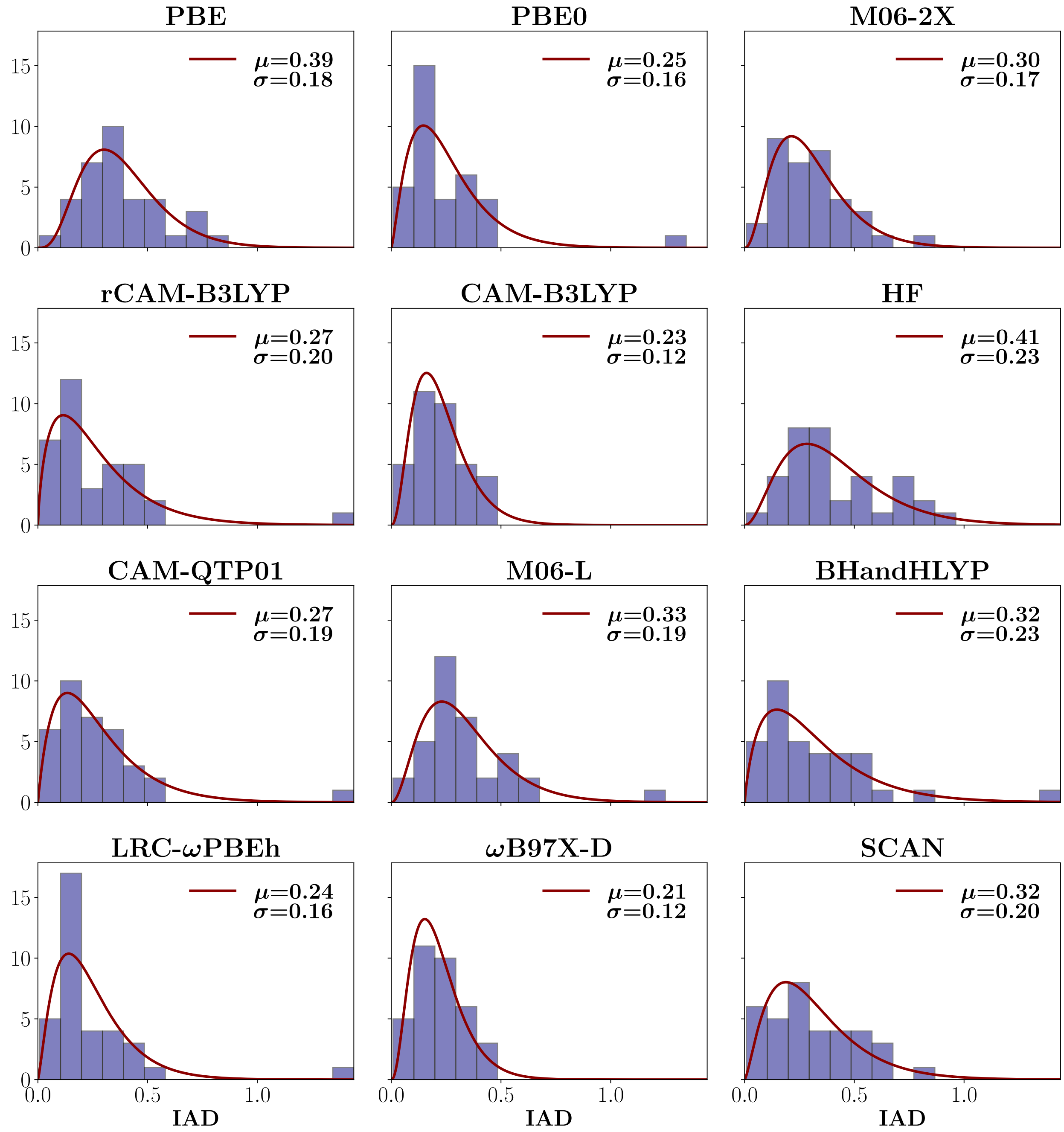

Figure S16: The distribution of optimally shifted IADs, on the **N 1s** resonance, in the **full-matrix diagonalization** approach.

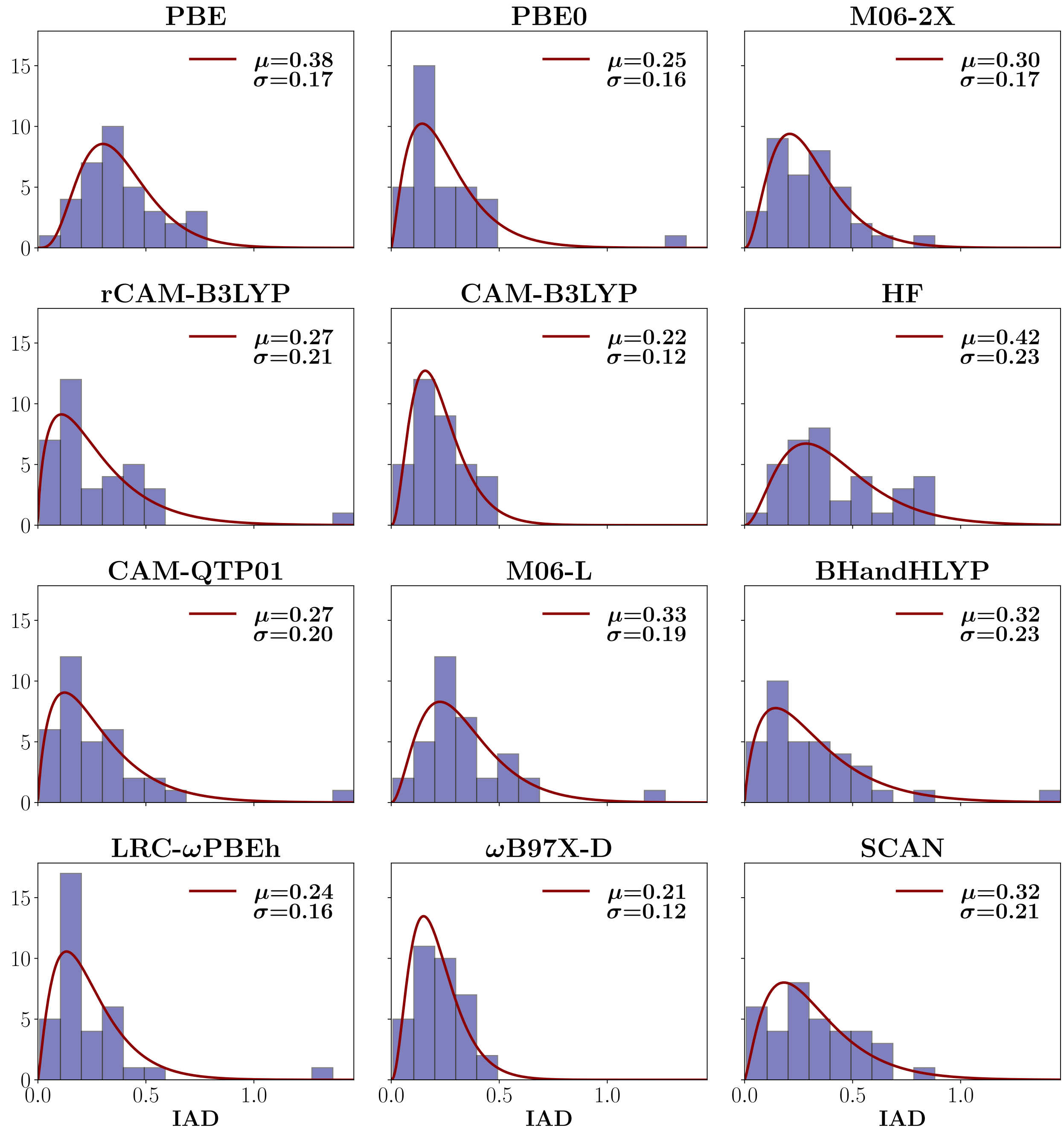

Figure S17: The distribution of optimally shifted IADs, on the **N 1s** resonance, in the **2S** approach.

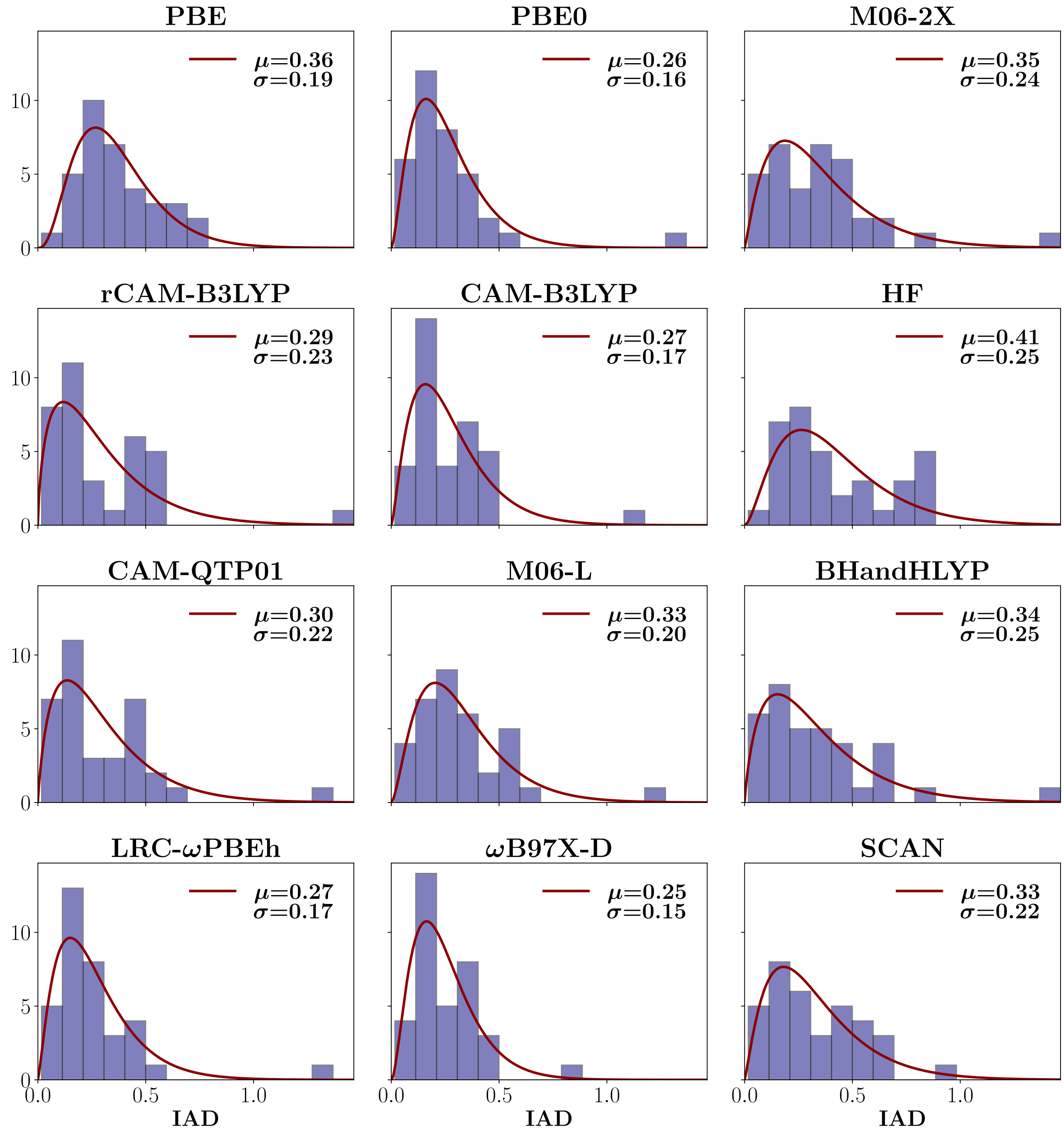

Figure S18: The distribution of optimally shifted IADs, on the **N 1s** resonance, in the **RSA** approach.

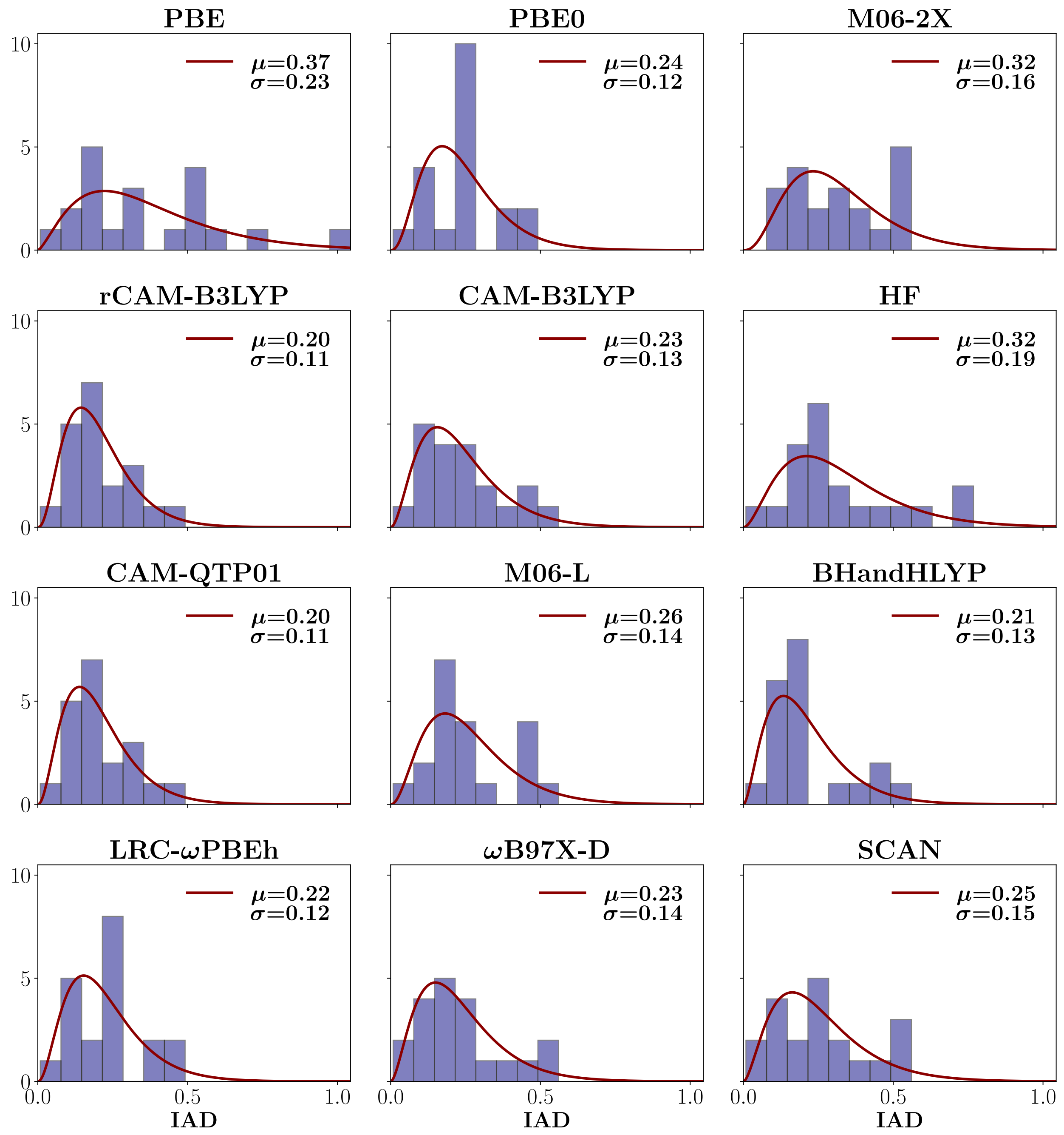

Figure S19: The distribution of optimally shifted IADs, on the **O 1s** resonance, in the **full-matrix diagonalization** approach.

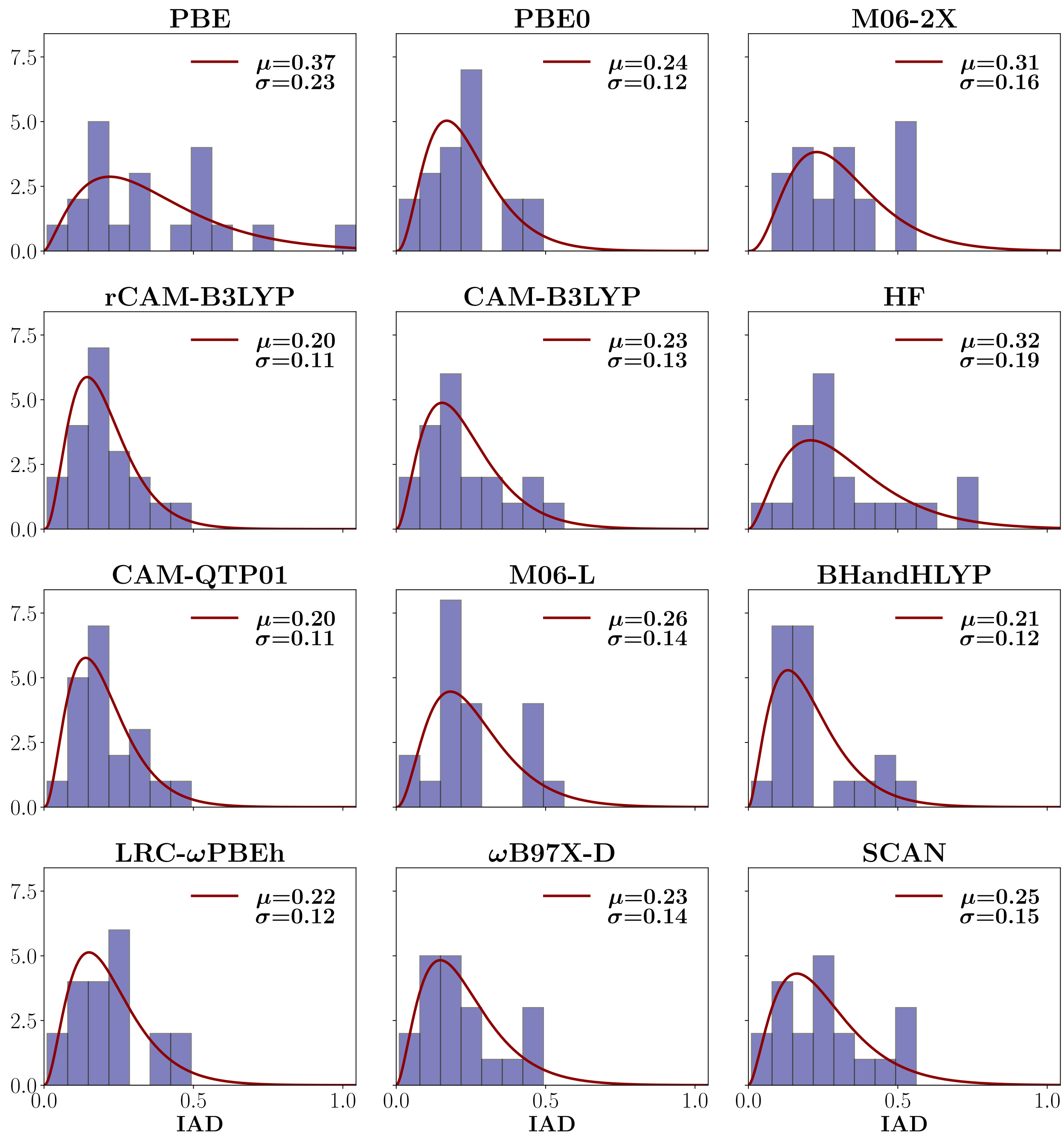

Figure S20: The distribution of optimally shifted IADs, on the **O 1s** resonance, in the **2S** approach.

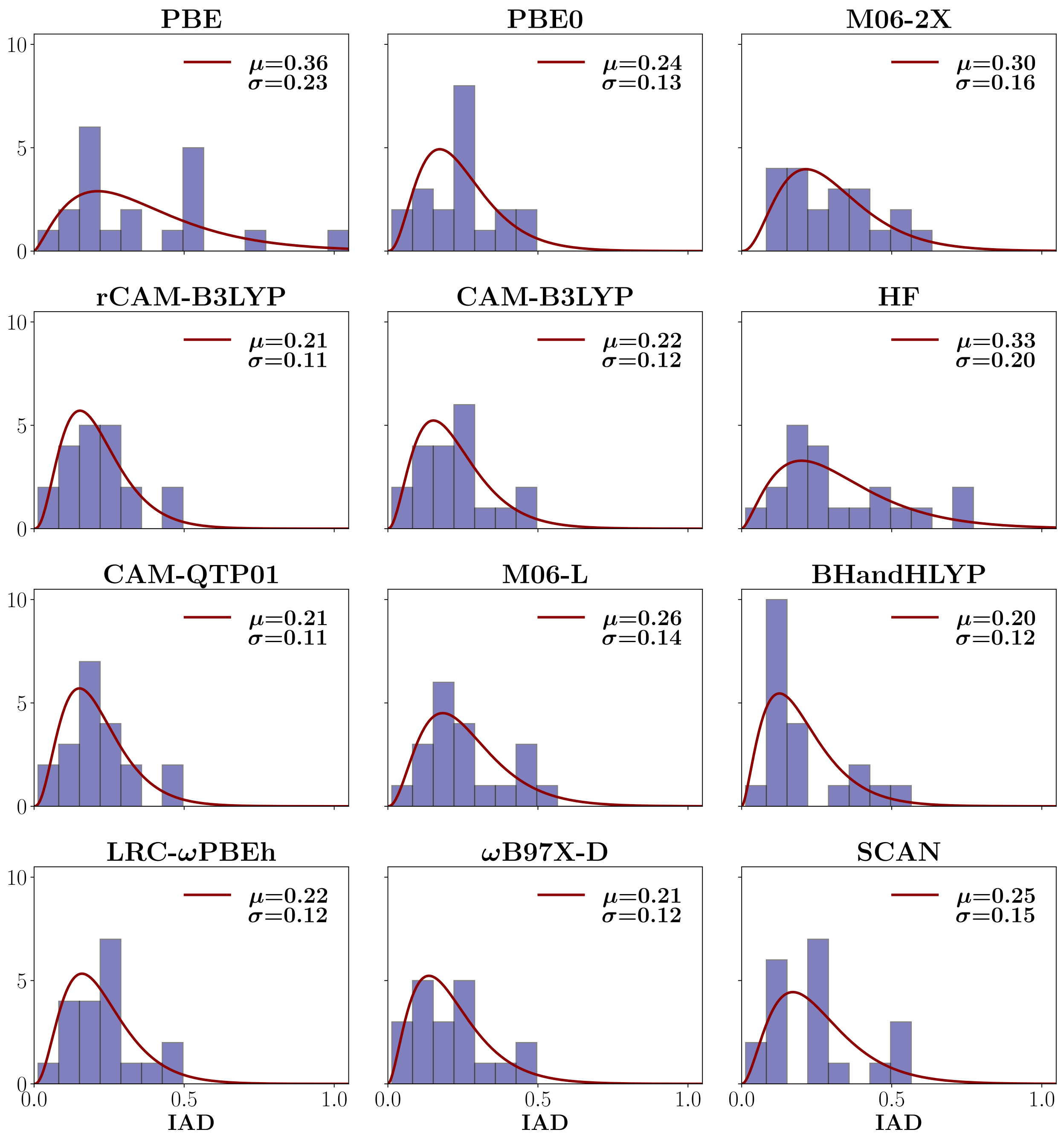

Figure S21: The distribution of optimally shifted IADs, on the **O 1s** resonance, in the RSA approach.

Mean Intensity Ratios

Table S2: RIXS mean intensity ratios of LR-TDDFT and 2S ADC for the first inelastic peak at the C K-edge.

Functional	Full Matrix	2S	RSA
PBE	1.05	1.01	1.09
PBE0	2.21	2.10	2.03
BHandHLYP	4.10	3.91	3.24
CAM-B3LYP	2.20	2.08	2.06
rCAM-B3LYP	2.33	2.18	2.14
CAM-QTP01	2.61	2.47	2.37
LRC-ωPBEh	2.08	1.91	1.89
ωB97X-D	2.44	2.32	2.22
SCAN	1.25	1.20	1.25
M06-L	1.36	1.29	1.35
M06-2X	3.14	3.00	2.72
HF	7.71	7.23	4.46

Table S3: RIXS mean intensity ratios of LR-TDDFT and 2S ADC for the first inelastic peak at the N K-edge.

Functional	Full Matrix	2S	RSA
PBE	0.67	0.65	0.69
PBE0	1.56	1.52	1.48
BHandHLYP	3.01	2.87	2.52
CAM-B3LYP	1.54	1.49	1.50
rCAM-B3LYP	1.67	1.62	1.63
CAM-QTP01	1.77	1.80	1.76
LRC-ωPBEh	1.42	1.37	1.37
ωB97X-D	1.62	1.57	1.55
SCAN	0.87	0.83	0.86
M06-L	0.72	0.69	0.72
M06-2X	2.53	2.49	2.27
HF	6.34	5.94	4.33

Table S4: RIXS mean intensity ratios of LR-TDDFT and 2S ADC for the first inelastic peak at the O K-edge.

Functional	Full Matrix	2S	RSA
PBE	1.06	1.03	1.11
PBE0	1.67	1.63	1.60
BHandHLYP	3.93	3.84	2.66
CAM-B3LYP	1.54	1.50	1.52
rCAM-B3LYP	1.54	1.49	1.51
CAM-QTP01	1.66	1.62	1.60
LRC-ωPBEh	1.49	1.46	1.46
ωB97X-D	1.61	1.57	1.57
SCAN	1.29	1.25	1.29
M06-L	1.14	1.10	1.16
M06-2X	3.67	3.60	3.38
HF	7.24	6.98	4.76

Effects of the Tamm-Dancoff approximation

In the table given below, the performance of the Tamm-Dancoff approximation (TDA) is compared to LR-TDDFT for the computation of RIXS. The comparison is made for the N K-edge using the rCAM-B3LYP functional in the 2S approach. The LR-TDDFT results are included for ease of comparison but are the same as found in Figs. (5-7) of the main text. As

Table S5: Performance of TDA compared to LR-TDDFT at the N K-edge with the rCAM-B3LYP functional for the three metrics: (optimally shifted) mean IAD, the mean absolute shift $|\Delta|$, and the mean intensity ratio of the first peak.

	IAD	Absolute Shift, $|\Delta|$ (eV)	Intensity, I/I_r
TDA	0.28	0.16	1.76
LR-TDDFT	0.27	0.17	1.62

it can be seen from the table, the error introduced by TDA is very small, especially in terms of the relative spectral features (IAD) and the energy position of the peaks ($|\Delta|$), while the absolute intensities are the most affected, where TDA overestimates the intensity somewhat more than LR-TDDFT.

Comparison between 2S and RSA

To determine the relative performance of the two LR-TDDFT approaches for RIXS, i.e. the 2S method and the RSA, we compare them to LR-TDDFT based on full matrix diagonalization. The diagonalization of the full response matrix corresponds to the exact solution within LR-TDDFT and therefore constitutes a reference to assess the errors introduced by neglecting the coupling elements between the core and valence manifolds in CVS (2S), working with possibly non-orthogonal core-excited and valence-excited states (2S), or restricting the donor and acceptor excitation spaces (RSA). For this comparison, the statistical metrics (IADs, absolute energy shifts, and intensity ratios) are evaluated using full-matrix diagonalization RIXS spectra as the reference. This represents the relative performance of the two approximate approaches 2S and RSA.

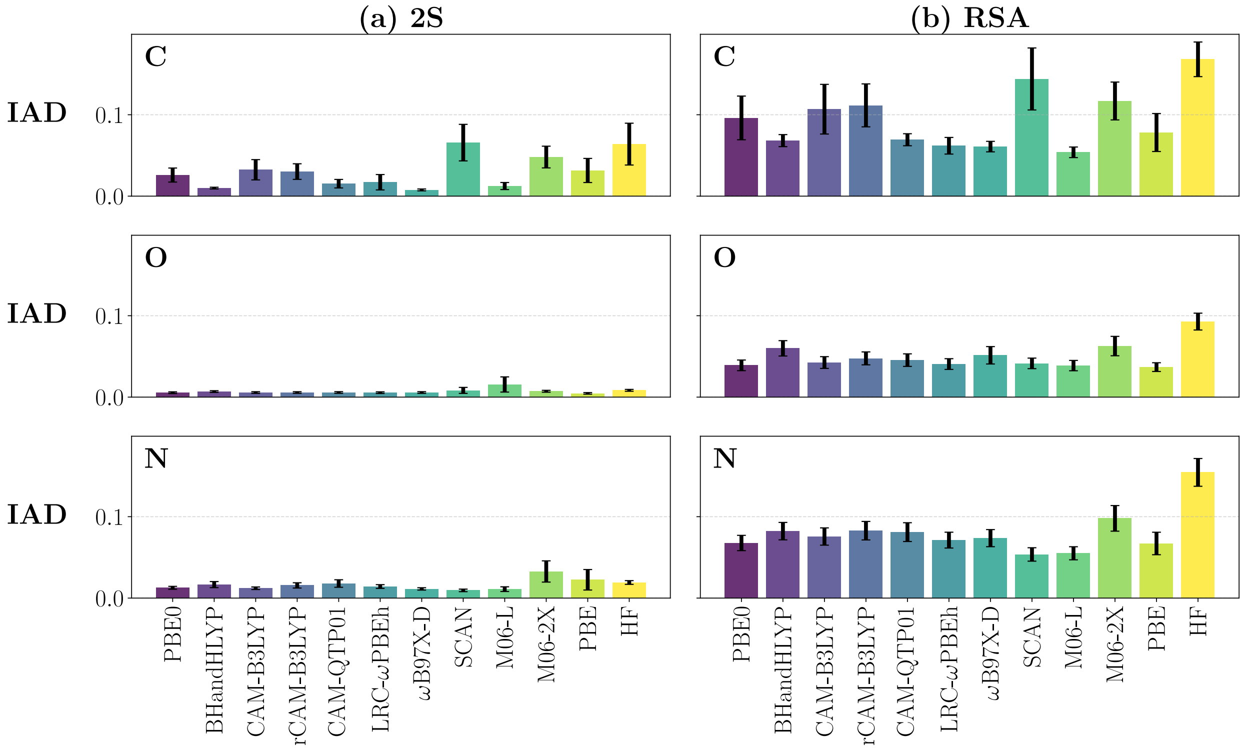

Figure S22: The distribution of optimally shifted IADs with full-matrix diagonalization as the reference method.

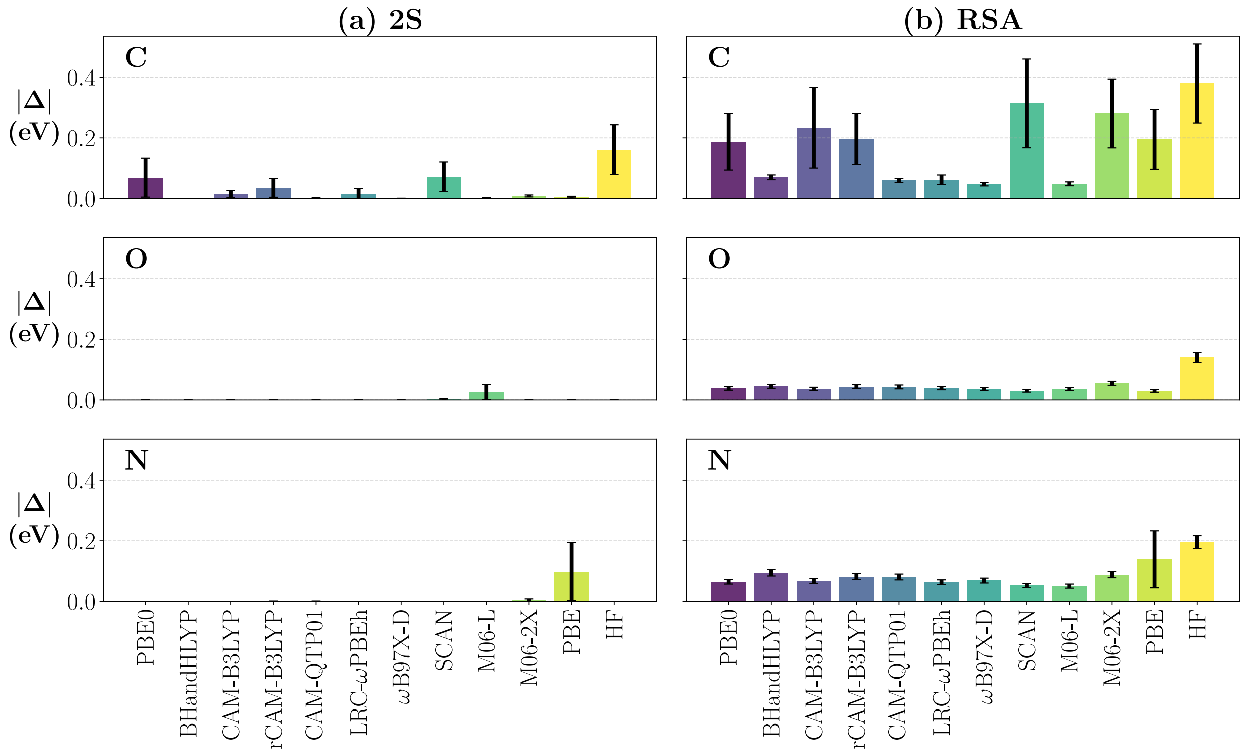

Figure S23: The distribution of absolute shifts with full-matrix diagonalization as the reference method.

The first observation from Figures S22-S24 is that the differences between 2S/RSA and full matrix diagonalization are very small, both in terms of relative spectral features, as well as absolute peak positions and intensities. The second observation is that 2S performs somewhat better than RSA, especially in terms of the absolute energy-shift metric.

The deviations seen in the 2S results of Figures S22 and S23 reflect errors arising due to the CVS approximation—that is, the decoupling of the core-excitations manifold from the valence. The trends in the 2S IADs and absolute shifts are consistent with the intuition that CVS is a better approximation when the core orbitals are energetically further separated from the valence. Accordingly, the errors are generally smallest for the O K-edge (highest binding energies –BEs–, $\sim$ 530 eV), increase for the N K-edge ($\sim$ 400 eV BE), and are largest for the C K-edge ($\sim$ 290 eV BE).

For RSA, the source of error is more difficult to isolate, as both the virtual and occupied spaces are truncated. Still, a similar trend emerges: the C K-edge exhibits the largest

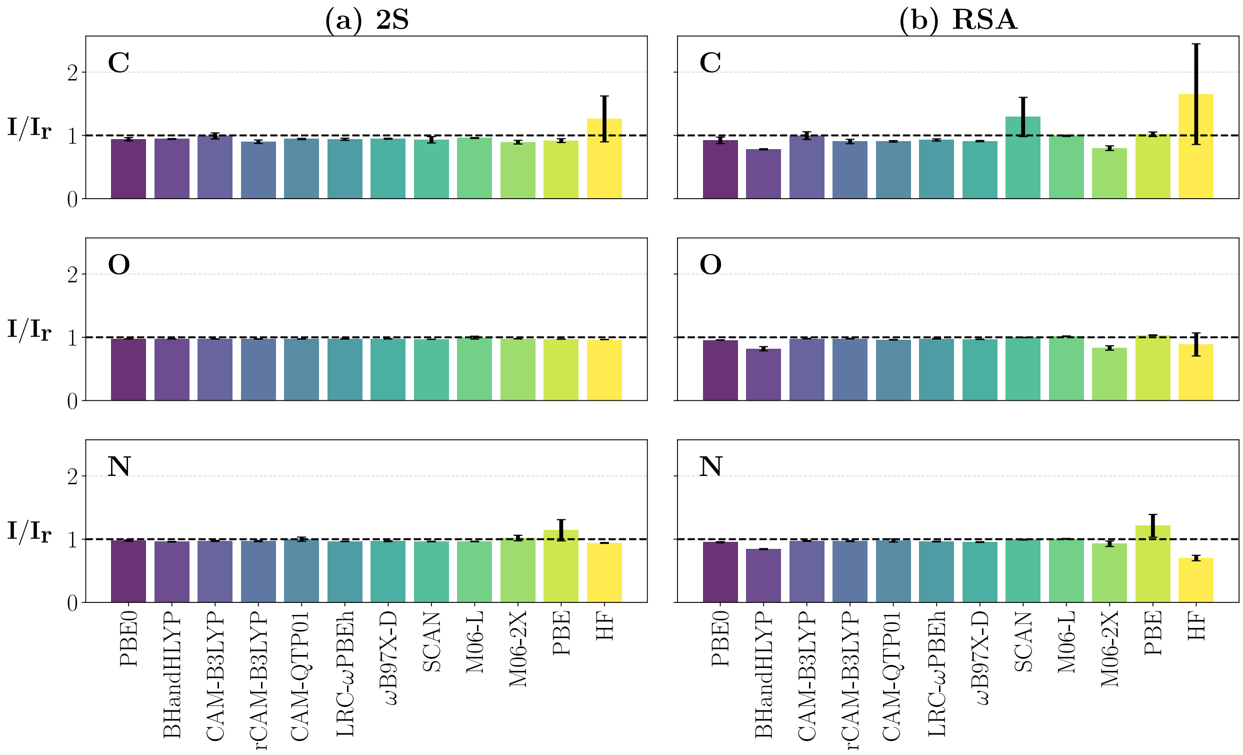

Figure S24: The distribution of intensity ratios with full-matrix diagonalization as the reference method.

deviations, followed by N and then O. In comparison to the CVS approximation, RSA does not completely decouple the core and valence manifolds, but it neglects the coupling with intermediate excited states or excitations to high energy unoccupied orbitals (i.e. the intermediate occupied orbitals between the core and valence, as well as high energy unoccupied orbitals are considered frozen and not included in the response matrix). The errors in RSA are evidently related to the choice on how to truncate the occupied and virtual spaces. Here, we included 35% of the virtual orbitals above the LUMO, while removing all the higher energy virtual MOs alongside 40% of the non-core occupied ones. However, other considerations of how to restrict the orbital space, e.g. based on energy thresholds rather than sheer number of orbitals, could provide better performance.

References

(1) Adamo, C.; Barone, V. Toward reliable density functional methods without adjustable parameters: The PBE0 model. *J. Chem. Phys.* **1999**, *110*, 6158–6170.

(2) Kendall, R. A.; Dunning, T. H.; Harrison, R. J. Electron affinities of the first-row atoms revisited. Systematic basis sets and wave functions. *J. Chem. Phys.* **1992**, *96*, 6796–6806.

(3) Boys, S. F. Construction of Some Molecular Orbitals to Be Approximately Invariant for Changes from One Molecule to Another. *Rev. Mod. Phys.* **1960**, *32*, 296–299.

(4) Weinhardt, L.; Weigand, M.; Fuchs, O.; Bär, M.; Blum, M.; Denlinger, J. D.; Yang, W.; Umbach, E.; Heske, C. Nuclear dynamics in the core-excited state of aqueous ammonia probed by resonant inelastic soft x-ray scattering. *Phys. Rev. B* **2011**, *84*, 104202.

(5) Weinhardt, L.; Benkert, A.; Meyer, F.; Blum, M.; Wilks, R. G.; Yang, W.; Bär, M.; Reinert, F.; Heske, C. Nuclear dynamics and spectator effects in resonant inelastic soft x-ray scattering of gas-phase water molecules. *J. Chem. Phys.* **2012**, *136*, 144311.

(6) Benkert, A.; Meyer, F.; Hauschild, D.; Blum, M.; Yang, W.; Wilks, R. G.; Bär, M.; Reinert, F.; Heske, C.; Weinhardt, L. Isotope Effects in the Resonant Inelastic Soft X-ray Scattering Maps of Gas-Phase Methanol. *J. Phys. Chem. A* **2016**, *120*, 2260–2267.

(7) Lehtola, S.; Steigemann, C.; Oliveira, M. J.; Marques, M. A. Recent developments in libxc — A comprehensive library of functionals for density functional theory. *SoftwareX* **2018**, *7*, 1–5.

(8) Rinkevicius, Z.; Li, X.; Vahtras, O.; Ahmadzadeh, K.; Brand, M.; Ringholm, M.; List, N. H.; Scheurer, M.; Scott, M.; Dreuw, A. et al. VeloxChem: A Python-driven density-functional theory program for spectroscopy simulations in high-performance computing environments. *WIREs: Comput. Mol. Sci.* **2020**, *10*, e1457.

(9) Perdew, J. P.; Burke, K.; Ernzerhof, M. Generalized gradient approximation made simple. *Phys. Rev. Lett.* **1996**, *77*, 3865.

(10) Becke, A. D. A new mixing of Hartree–Fock and local density-functional theories. *J. Chem. Phys.* **1993**, *98*, 1372–1377.

(11) Yanai, T.; Tew, D. P.; Handy, N. C. A new hybrid exchange–correlation functional using the Coulomb-attenuating method (CAM-B3LYP). *Chem. Phys. Lett.* **2004**, *393*, 51–57.

(12) Cohen, A. J.; Mori-Sánchez, P.; Yang, W. Development of exchange-correlation functionals with minimal many-electron self-interaction error. *J. Chem. Phys.* **2007**, *126*, 191109.

(13) Jin, Y.; Bartlett, R. J. The QTP family of consistent functionals and potentials in Kohn-Sham density functional theory. *J. Chem. Phys.* **2016**, *145*, 034107.

(14) Rohrdanz, M. A.; Martins, K. M.; Herbert, J. M. A long-range-corrected density functional that performs well for both ground-state properties and time-dependent density functional theory excitation energies, including charge-transfer excited states. *J. Chem. Physics* **2009**, *130*, 054112.

(15) Chai, J.-D.; Head-Gordon, M. Long-range corrected hybrid density functionals with damped atom–atom dispersion corrections. *Phys. Chem. Chem. Phys.* **2008**, *10*, 6615–6620.

(16) Zhao, Y.; Truhlar, D. G. A new local density functional for main-group thermochemistry, transition metal bonding, thermochemical kinetics, and noncovalent interactions. *J. Chem. Phys.* **2006**, *125*, 194101.

(17) Zhao, Y.; Truhlar, D. G. The M06 suite of density functionals for main group thermochemistry, thermochemical kinetics, noncovalent interactions, excited states, and

transition elements: two new functionals and systematic testing of four M06-class functionals and 12 other functionals. *Theor. Chem. Acc.* **2008**, *120*, 215–241.

(18) Sun, J.; Ruzsinszky, A.; Perdew, J. P. Strongly Constrained and Appropriately Normed Semilocal Density Functional. *Phys. Rev. Lett.* **2015**, *115*, 036402.
